# Three potential neurovascular pathways driving the benefits of mindfulness meditation for older adults

**DOI:** 10.3389/fnagi.2023.1207012

**Published:** 2023-06-29

**Authors:** Jessica Pommy, Colette M. Smart, Andrew M. Bryant, Yang Wang

**Affiliations:** ^1^Department of Neurology, Division of Neuropsychology, Medical College of Wisconsin, Milwaukee, WI, United States; ^2^Department of Psychology, University of Victoria, Victoria, BC, Canada; ^3^Department of Neurology, The Ohio State University, Columbus, OH, United States; ^4^Department of Radiology, Medical College of Wisconsin, Milwaukee, WI, United States

**Keywords:** mindfulness meditation, functional MR (fMRI), aging, neurovascular aging, neurobiological mechanism of action

## Abstract

Mindfulness meditation has been shown to be beneficial for a range of different health conditions, impacts brain function and structure relatively quickly, and has shown promise with aging samples. Functional magnetic resonance imaging metrics provide insight into neurovascular health which plays a key role in both normal and pathological aging processes. Experimental mindfulness meditation studies that included functional magnetic resonance metrics as an outcome measure may point to potential neurovascular mechanisms of action relevant for aging adults that have not yet been previously examined. We first review the resting-state magnetic resonance studies conducted in exclusively older adult age samples. Findings from older adult-only samples are then used to frame the findings of *task* magnetic resonance imaging studies conducted in both clinical and healthy adult samples. Based on the resting-state studies in older adults and the task magnetic resonance studies in adult samples, we propose three potential mechanisms by which mindfulness meditation may offer a neurovascular therapeutic benefit for older adults: (1) a direct neurovascular mechanism via increased resting-state cerebral blood flow; (2) an indirect anti-neuroinflammatory mechanism via increased functional connectivity within the default mode network, and (3) a top-down control mechanism that likely reflects both a direct and an indirect neurovascular pathway.

## 1. Introduction

There is growing interest in the application of mindfulness meditation (MM) for the management of symptoms associated with neurological conditions (Pickut et al., 2013; Smart et al., 2016, 2021; Smart and Segalowitz, 2017; Bachmann et al., 2018; Poissant et al., 2019; Shim et al., 2020). Briefly, MM encompasses multiple practices, all of which emphasize intentionally becoming aware of and directing one’s attention to specific aspects of one’s present moment experience non-judgmentally (Kabat-Zinn et al., 1985). The literature on neural/cognitive changes in healthy populations presents a foundation on which to explore the application to persons with neurological disorders (Smart, 2019). A recently published review examined the benefits of MM across a range of neurocognitive conditions and found MM showed promise for neurodegenerative disorders, with moderate to large effect sizes in studies that included neurological biomarkers as outcome measures (Smart et al., 2021). In older adults, MM has been associated with better cognitive abilities [particularly, memory (Berk et al., 2017; Hazlett-Stevens et al., 2019), executive function (Berk et al., 2017; Hazlett-Stevens et al., 2019), and processing speed (Berk et al., 2017)] and was hypothesized to boost cognitive reserve (Fountain-Zaragoza and Prakash, 2017; Malinowski and Shalamanova, 2017). Additionally, MM has been linked to improved mood (Gallegos et al., 2013; Geiger et al., 2016; Li and Bressington, 2019) and increased engagement in pro-health behaviors (Nagaoka et al., 2021). As neuroimaging methods are more sensitive to changes associated with aging relative to measures of cognitive function, and frequently, are the earliest indicators of pathological aging (before symptom onset) (Bertens et al., 2015; Hampel et al., 2021; Petersen et al., 2021), these methods are of interest for understanding the potential therapeutic mechanisms of actions of MM for older adults.

Previous review articles have focused primarily on mechanisms derived from structural magnetic resonance (MR) studies (Epel et al., 2009; Prakash et al., 2014; Laneri et al., 2015; Larouche et al., 2015; Fountain-Zaragoza and Prakash, 2017; Ramirez-Barrantes et al., 2019). Indeed, there is a compelling body of literature linking MM to structural MR changes that are relevant for both normal and pathological aging. Early studies of MM in older adults, for instance, examined structural brain changes in cohorts of long-term MM practitioners and found reduced age-related brain volume loss among older adults with years of regular MM practice (Luders et al., 2009, 2012, 2013a,b, 2014, 2021; Kurth et al., 2015). Subsequent research demonstrated it was possible to induce similar changes on structural MR experimentally over a much briefer timeframe (e.g., 8 weeks) (Tang et al., 2010, 2012, 2019; Laneri et al., 2015). Functional MR may elucidate alternative, novel therapeutic mechanisms driving the benefits of MM for older adults.

Functional MR [e.g., Blood-Oxygen-Level-Dependent (BOLD) signal and arterial spin labeling (ASL) measurements] are frequently used as a means for estimating neurovascular function. Increasingly, neurovascular processes are being recognized as integral to our understanding of neurodegeneration in both normal and pathological aging (Du et al., 2017; Tarantini et al., 2017; Abdelkarim et al., 2019; Korte et al., 2020; Tsvetanov et al., 2021). Changes in neurovascular function have been detected *before* onset of gray matter volume loss in early stage Alzheimer’s Disease (AD) (Korte et al., 2020; Nielsen et al., 2020) and mechanistically, neurovascular changes may be a driving mechanism underlying normative age-related volume loss as well (Abdelkarim et al., 2019; Turner, 2021). To our knowledge no review paper yet published has focused on functional MR metric changes as a means for understanding the benefits of MM in older adults framed via a neurovascular mechanism.

The primary goal of this paper is to: (1) review MM intervention studies that included functional MR (task and resting-state) as an outcome measure and conceptualize these findings within the broader aging and neurodegenerative literature, and (2) consider how these findings may provide support for a potential neurovascular pathway that could drive the therapeutic benefits of MM for older adults. To provide the reader with the motivation for the current review and to provide the necessary context for our subsequent framing of functional MR studies, we will begin with a brief summary of the key neurovascular concepts relevant for functional MR, aging, and MM.

### 1.1. Background and key concepts

#### 1.1.1. Mindfulness meditation

Mindfulness meditation (MM) is operationalized in a variety of ways in the literature. For example, researchers have examined the effects of brief state-based manipulations, standardized programs like Mindfulness-Based Stress Reduction (MBSR) or Mindfulness-Based Cognitive Therapy (MBCT), and have compared novice and expert meditators (i.e., long-term meditators) on various outcome measures (Smart, 2019). Further, there is a significant cross-sectional body of literature comparing long-term (LT) meditators to matched healthy adults with no meditation experience (Luders et al., 2009, 2013b,^2014^, ^2015^; Newberg et al., 2010; Kurth et al., 2015; Laneri et al., 2015). Experimental studies have demonstrated the effects of MM on the brain can emerge over relatively brief timeframes (e.g., 8-week intervention studies to 15 min MM induction) (Smart, 2019). For instance, increased functional connectivity in resting-state networks was reported following 2 weeks of MM (Tang and Posner, 2014), while increased resting-CBF was reported after 5 days of MM (Tang et al., 2015). Finally, the benefits of MM have been demonstrated in both healthy samples and clinical samples, with some evidence to suggest the effects may be particularly robust within clinical samples (Keng et al., 2011).

#### 1.1.2. Functional MR terms

Functional MR studies that employ a task-based approach examine signal changes in BOLD (or ASL) during performance of a given task (e.g., performance of a cognitive task, passive viewing of stimuli) relative to a preidentified baseline or rest condition (Soares et al., 2016). In contrast, resting-state functional MR examines BOLD (or ASL) in the absence of any task. Functional connectivity analyses are a frequently used analysis approach that examines the temporal coherence of different brain regions via large network analyses (e.g., seed-based, independent component, graph theory) (Rosazza and Minati, 2011; Soares et al., 2016) and can be applied to resting-state or task MR. Several reliable brain networks across populations have emerged (Raichle et al., 2001), including the Default Mode Network (DMN) (Raichle et al., 2001; Uddin et al., 2009), as well as resting state networks related to cognitive functions, including the Dorsal and Ventral Attention Networks, Cognitive Control Network, and Salience Network, among others (Raichle et al., 2001; Rosazza and Minati, 2011; Raichle, 2015; Uddin et al., 2019). While task-based approaches point to how the brain is functioning under specific cognitive demands [e.g., increased CBF during cognitively demanding tasks observed with increasing age (Langenecker and Nielson, 2003; Vallesi et al., 2011)], resting-state approaches may reflect condition specific phenotypes (Rosazza and Minati, 2011) [e.g., reduced connectivity within resting-state networks observed in Alzheimer’s Disease (Dillen et al., 2017; Ibrahim et al., 2021)], are associated with aging (Song et al., 2012), and are typically reliable within-individuals (Shehzad et al., 2009; Mennes et al., 2010).

#### 1.1.3. Functional MR relies on neurovascular function

Functional MR methods are dependent on the phenomena of neurovascular coupling (NVC), i.e., the regional increase in cerebral blood flow (CBF) following neuronal firing that supplies that brain region with glucose and oxygen (Kim and Filosa, 2012). NVC relies upon a responsive vasculature system and unsurprisingly, declines in vascular health, and in particular, the health of the brain’s vasculature have a detrimental impact on NVC (Yu et al., 2020). When there is a reduction in the ability of the cerebral blood vessels to dilate and constrict in response to vasoactive stimuli (also known as cerebrovascular reactivity; CVR) (Magon et al., 2009), for instance, reductions in resting CBF and/or neurovascular uncoupling can be observed (Kisler et al., 2017a,b). To capture the complex interaction between the vascular system and the brain, the neurovascular unit (NVU) was proposed (Yu et al., 2020; Naranjo et al., 2022; Nizari et al., 2022). Briefly, the NVU is a complex and interacting system that underlies regulation of CBF and maintenance of the blood brain barrier. The NVU is comprised of vascular cells (particularly, pericytes, and endothelial cells), glial cells (astrocytes and microglial) and neurons that form a complex feedback loop (Schaeffer and Iadecola, 2021). Disruptions to the NVU can trigger a multitude of metabolic and inflammatory processes that ultimately are detrimental for NVC (e.g., reactive oxidative stress, altered nitric oxide pathways) (Hosford and Gourine, 2019; Hoiland et al., 2020; Zhu et al., 2021). While the vast majority of research has focused on the NVU at the microvascular level, these processes can be studied across different levels of analysis as well (e.g., inflammatory markers linked to cognitive declines in older adults and reduced NVC) (Naranjo et al., 2022; Nizari et al., 2022). Changes on functional MR metrics in normal and pathological aging, may, in part be driven by changes in the NVU (Yu et al., 2020; Naranjo et al., 2022; Nizari et al., 2022).

#### 1.1.4. MM impacts measures of vascular health and components of the NVU

Mindfulness meditation has been linked to improvements in cardiovascular health among older adults at risk for cerebrovascular disease (Marino et al., 2021), with studies showing improvements in cholesterol (Gentile et al., 2021), blood pressure (Palta et al., 2012; Marino et al., 2021), heart rate variability and heart rate by age (Chang et al., 2020). The mechanisms driving the benefits of MM for cardiovascular health are likely multifactorial. A recent review article highlighted the potential for MM to improve psychological factors (depression, anxiety, and stress) in samples with cardiovascular health problems which in turn improved markers of vascular health (Marino et al., 2021). Similarly, efforts have been made to link changes in the stress response following MM and improved vascular health [e.g., MM reduced skin conductance response and high-frequency heart rate variability, both markers of the parasympathetic response (Tang et al., 2020) and reduced cortisol awakening response, also linked to heart health (Chouinard et al., 2019)].

Mindfulness meditation has also been shown to impact vascular function more directly across several different body systems (e.g., improved myocardial oxygen consumption (Kim et al., 2017), improved optic disc perfusion and vessel density (Dada et al., 2022), and decreased intraocular pressure (Dada et al., 2018, 2021a,b; Gagrani et al., 2018; Dada and Gagrani, 2019). Of interest for NVU health, MM was linked to improved endothelial function (Kim et al., 2017) as well as, decreased inflammation and decreased reactive oxidative stress (Gagrani et al., 2018). Interestingly, the latter process (decreased reactive oxidative stress) was also found to correlate with increased blood flow at rest in the prefrontal cortex as measured using functional-near infrared spectroscopy (Gagrani et al., 2018), providing initial support linking these mechanisms more directly to neurovascular function. Results from DNA methylation studies provide further support. In particular, MM was associated with improved endothelial function and cellular metabolism (via upregulation of the neuroglobin gene which is involved in oxygen transport and mitochondrial functioning, reduced oxidative stress, and changes to the nitric oxide synthetase system) (Dada et al., 2018, 2021a) in older adults and also impacted genes linked to neuroinflammation and glial activation (Dada et al., 2021a) relevant in AD.

## 2. Literature search

A literature search was conducted using Web of Science Topic search (words included in title, abstract, or key words submitted by author) option to identify all task and resting-state fMRI studies. For the task fMRI review, we searched all research articles using “Task fMRI” AND: “Mindfulness” Or “Mindfulness-based” OR “meditation.” For the resting-state fMRI review, we examined all articles using topic search: “resting-state networks” OR “resting-state fMRI” AND “mindfulness meditation” OR “mindfulness based” OR “mindfulness” OR “meditation.” We additionally ran a second search specifically targeting the default mode network: topic: “default mode network” AND “mindfulness based” OR “meditation.” Only articles with full text were included (conference abstracts were excluded). For the purposes of this review, we will focus on experimental studies of MM that have included task or resting-state fMRI as outcome measures. Papers that focused exclusively on yoga or transcendental meditation and did not include MM practices *per se* (e.g., use of focused attention or open monitoring practices) were excluded as these practices would rely more heavily on speech and motor networks (Fox et al., 2016). Additionally, trait mindfulness studies, MM dismantling studies and studies that did not use a facilitator (e.g., participants listened to recording without any interaction or tailoring to individual) were excluded. The remaining study abstracts were read to assess fit. Review and opinion papers were excluded. Additionally, studies that used exclusively alternate neuroimaging methods (e.g., EEG, PET) and studies that examined only meditation-like tasks without an intervention/training component were excluded. Interventions that focused on child or adolescent samples, or exclusively on undergraduate age students (18–22 years of age or mean age less than 25 years of age; or studies that included no age information) were excluded. Results from studies conducted in older adults (age 60 or older) were examined first. Results from those studies were used to frame findings from younger and middle-aged adult samples.

## 3. Results

Overall, our search identified 55 articles that included functional MR as an outcome measure (38 of 55 articles reported resting-state MR; 18 of 55 articles reported task functional MR) and met inclusion criteria. Results were organized based on age and outcome measure. Seven of the 55 articles focused exclusively on an older adult sample and examined changes in resting-state MR. None of the task functional MR studies were conducted in exclusively an older adult sample.

A total of 7 articles examined resting-state MR (7 articles reported BOLD (Wells et al., 2013; Shao et al., 2016; Cotier et al., 2017; Fam et al., 2020; Sevinc et al., 2021; Li et al., 2022; Moss et al., 2022) and 1 of 7 also reported ASL (Moss et al., 2022) as an outcome measure following MM in an older adult sample (age 60 and older). The 7 resting-state MR articles will be reviewed first (along with one additional ASL study that focused primarily on an older adult sample but included middle-aged adults as well). Findings from the resting-state MR studies in older adults will be used to frame the review of the 18 task functional MR studies. Additional details regarding methods including intervention type and duration of MM of the articles reviewed within the main text are provided in Supplementary Table 1. The results from the 30 resting-state MR articles conducted using younger and middle-aged adults are summarized in Supplementary Table 2 using a similar framing as the task functional MR studies.

### 3.1. Prolonged effects of MM in cognitively unimpaired older adults

Four articles (3 studies) were conducted with a healthy aging sample (Shao et al., 2016; Cotier et al., 2017; Sevinc et al., 2021; Moss et al., 2022), Finally, one additional article that reported ASL changes in a combined middle-aged and older adult sample (age range: 52–77) (Monti et al., 2012) and will be discussed in conjunction with the single older adult ASL study (Moss et al., 2022). Regarding sample demographics, only 1 of the 7 resting-state MR articles reported data on the sample’s race, 1 of 7 reported on the sample’s ethnicity, 6 of the 7 studies reported on sex of the sample, 4 of the 7 reported education level, and 0 of the 7 studies reported on socioeconomic status (SES).

#### 3.1.1. Changes in resting-state ASL in cognitively unimpaired older adults

Moss et al. (2022) examined changes in resting-state CBF in healthy older adults (71–87 years old, mean age of 79) following MM (pre/post design). More specifically, using an asymmetric pulsed ASL sequence, Moss et al. (2022) reported increased resting-state CBF in right prefrontal cortex, anterior cingulate, posterior cingulate, left superior temporal gyrus and left superior frontal gyrus (Moss et al., 2022). Results from the functional connectivity analyses using BOLD will be discussed in the following section. A second study used continuous and pseudo-continuous ASL techniques, Monti et al. (2012) reported increased resting state CBF in bilateral caudate and left insula following MM (compared to the control group) among older women (52–77 years of age) with breast cancer. Within-group analyses of the MM group showed additional increases in CBF following intervention in right amygdala, hippocampus, middle temporal cortex, as well as, left insula, and bilateral caudate and fusiform gyrus/cerebellum, with no increases observed in the control group. Further, within the MM group there was a significant pre/post drop in anxiety which was correlated with increased resting-state CBF in the left caudate. Results from these studies are provided in Table 1.

**TABLE 1:** Prolonged effects of MM on resting-state ASL in cognitively unimpaired older adults.

References	Age range	Demographics	Within-group MM effect^A^	Group by time interaction^B^	Regions	Behavioral correlates in MM
Moss et al., 2022	71–87	*N* = 11 (9f, 2 m) Age = 79 (5.2) Edu: 18 (3) 11 W No information for SES or ethnicity.	Pre < Post		R pfc, acc, pcc, L stg, L sfg	
Monti et al., 2012	52–77	MM: *N* = 8 (8f, 0 m) Age = 56 (7) 2 AA 7 W AC: *N* = 10 (10f, 0 m) 3 AA 1 A 6 W No information for ethnicity, edu, or SES.	Pre < Post	MM > AC	B caudate L insula R amygdala, R hipp, B mtg, L insula/inferior frontal, B caudate, B fusiform/occipital/ cerebellum	Decreased anxiety correlated with increased L caudate CBF.

The values within Age Range column reflect age range of the study sample in years. Within the Demographics column, mean years of education and age are provided with standard deviation in parentheses when data was available. Race is abbreviated and the number reflects the corresponding sample sizes. A: This column is included for studies that demonstrate a significant effect of time within the MM group. Pre < Post reflects a within-group contrast with greater ASL at the follow up visit (after MM intervention) compared to baseline scan. B: This column is included for studies in which an ANOVA was performed and demonstrated a significant treatment-by-time interaction (where treatment is MM or AC and time is pre or posttreatment). MM > AC reflects greater ASL in MM compared to AC after the intervention. ACC, anterior cingulate cortex; CBF, cerebral blood flow; Hipp, hippocampus; MTG, middle temporal gyrus; PCC, posterior cingulate cortex; PFC, prefrontal cortex; SFG, superior frontal gyrus; STG, superior temporal gyrus; AC, active control; AA, African American; A, Asian; B, bilateral; Edu, education; F, female; L, left; M, male; MM, mindfulness meditation; N, sample size; R, right; SES, socioeconomic status; W, white.

#### 3.1.2. Changes in resting-state networks in cognitively unimpaired older adults

Seven articles (derived from 6 studies) examined changes in resting-state functional connectivity (rsFC) associated with MM in older adults (Wells et al., 2013; Shao et al., 2016; Cotier et al., 2017; Fam et al., 2020; Sevinc et al., 2021; Li et al., 2022; Moss et al., 2022). There was notable heterogeneity in the methodology used across studies. Two of the seven articles examined cognitive changes and two studies examined emotion changes (mood/anxiety or emotional arousal).

Four of the seven articles examined changes within healthy aging adults (see Table 2). Shao et al. (2016) and Cotier et al. (2017) both examined the effects of MM relative to relaxation training within the same sample of healthy older adults (mean age approximately 65). Shao used a seed-based approach (posterior cingulate cortex, PCC) to examine whole-brain rsFC. Increased rsFC was found between the pons and PCC at follow-up within the MM group (significant group-by-time interaction). Follow up analyses revealed rsFC changes were associated with changes in emotional processing (reduced arousal and valence after the MM intervention). In a follow up paper, Cotier et al. (2017) examined the topology of the resting-state networks using graph metrics across levels of analysis (global connectivity, intra-module and inter-module, and nodal levels). Within the MM group, indicators of network efficiency improved at follow up within the MM group when examined at intermediate and local levels of analysis in the default mode network (DMN) and salience network (SN) (among other networks). Additionally, lower nodal strength was observed in key DMN regions as well (left PCC, bilateral paracentral lobule, and middle cingulate gyrus).

**TABLE 2:** Prolonged effects of MM on resting-state FC in cognitively unimpaired older adults.

References	Age range	Demographics	Within-group MM effect^A^	Group by time interaction^B^	Regions^C^	Behavioral correlates in MM.
Sevinc et al., 2021	65–80	MM: *N* = 70 (55.7%f) Age = 70.2 (4.1) Edu = 16.7 (1.8) AC: *N* = 75 (53.3%f) Age = 71.0 (4.3) Edu = 16.7 (1.9) No information provided for race, ethnicity, or SES.	Pre < Post (from gPPI)	NS	R hipp-R precuneus L hipp-R angular gyrus	Improved PACC scores at follow up correlated with increased R hipp-R precuneus and L hipp-R angular gyrus
Shao et al., 2016*	60–69	MM: *N* = 23 (16f, 7 m) Age = 64.78 (2.71) AC: *N* = 22 (14f, 6 m) Age = 64.68 (2.19) No information provided for edu, SES, race, or ethnicity.		MM > AC	Pons-pcc/precuneus (pons to pcc/precuneus based on effective connectivity)	Pons-pcc/precuneus positively correlated with reductions in task valence/arousal ratings.
Moss et al., 2022	71–87	*N* = 11 (9f, 2 m) Age = 79 (5.2) Edu: 18 (3) 11 W No information provided for SES or ethnicity.	Pre < Post Pre > Post		acc- L insula, pcc, putamen, and L ofc. Amygdala- R parahipp, R itg, R ofc, and R hipp. Parahipp- R hipp, R insula, R itg, and cerebellum. pfc- R stg, R itg. R amygdala- L hipp. R hipp-R parahipp L caudate nuclei-R caudate nuclei acc-cerebellum, L itg, L stg	
**Graph metrics**						
Cotier et al., 2017*	60–69	MM: *N* = 23 (16f, 8 m) Age = 64.78 (2.71) AC: *N* = 22 (14f, 8 m) Age = 64.68 (2.19) No information for race, ethnicity, edu, or SES.		MM < AC (intra-module) MM < AC (inter-module) MM < AC (Nodal Strength)	DMN-DMN, SN-SN, SMN-SMN DMN-SN, DMN-SMN L pcc, bilateral paracentral lobule, midCC	

An asterisk indicates two articles used the same sample. The values within the Age Range column reflect the age range of the study sample in years. Within the Demographics column, mean years of education and age are provided with standard deviation in parentheses when data was available. Race is abbreviated and the number reflects the corresponding sample sizes. A: This column is included for studies that demonstrate a significant effect of time within the MM group. Pre < Post reflects a within-group contrast with increased FC at the follow up visit (after MM intervention) compared to baseline (before MM intervention). Pre > Post reflects a within-group contrast with reduced FC at the follow up visit (after MM intervention) compared to baseline scan. B: This column is included for studies in which an ANOVA was performed and demonstrated a significant treatment-by-time interaction (where treatment is MM or AC and time is pre or posttreatment). NS reflects instances where a group × time interaction was examined but was not statistically significant (in which case no regions will be listed in the regions column). MM > AC reflects increased FC in MM compared to AC after the intervention. MM < AC reflects reduced FC in MM compared to the AC after the intervention. C: This column includes corresponding significant regions of the brain associated with contrast in A or B. A dash is used to indicate a connection between two regions. ACC, anterior cingulate cortex; DMN, default mode network; Hipp, hippocampus; ITG, inferior temporal gyrus; OFC, orbitofrontal cortex; midCC, middle cingulate gyrus; parahipp, parahippocampus; PCC, posterior cingulate cortex; pfc, prefrontal cortex; RSP, retrosplenial cortex; SN, salience network; STG, superior temporal gyrus; SMN, somatomotor network; AC, active control; B, bilateral; Edu, education; F, female; gPPI, generalized psychophysiological interaction; L, left; M, male; MM, mindfulness meditation; NS, not significant; PACC, Preclinical Alzheimer Cognitive Composite; R, right; SES, socioeconomic status; N, sample size.

Alternatively, using a within-subject design Moss et al. (2022) performed several regions of interest (ROI) analyses in a small sample of older adults (mean age of 79) (Moss et al., 2022). Increased rsFC was observed between multiple subcortical brain regions associated with memory and between the prefrontal cortex (PFC) and temporal regions (amygdala, hippocampus, parahippocampus). While the vast majority of connections lateralized to the right hemisphere (e.g., right hippocampus to right parahippocampus), increased rsFC was also observed interhemispherically (e.g., right amygdala-left hippocampus). Finally, both increased and decreased rsFC was reported for the ACC.

Sevinc et al. (2021) employed a different analysis approach to examine MM changes that might explain the cognitive benefits for healthy older adults (mean ages 70 and 71). Using a generalized psychophysiological interaction (gPPI) analysis, the authors narrowed the analyses to regions that explained cognitive differences within the sample prior to starting the intervention. Within the MM group (but not the control group) at follow up, there was increased rsFC between (1) right precuneus and right hippocampus; and (2) left hippocampus and right angular gyrus, both of which correlated with improved cognition at follow-up in the MM group. Of note, the initial seed-based rsFC analyses based on key regions of interest (posteromedial cortex, including PCC/retrosplenial cortex, and hippocampi seeds) did not show significant group by time interaction effects.

#### 3.1.3. Changes in resting-state networks in older adults with cognitive or behavioral changes

Two articles were conducted within a mild cognitive impairment sample (Wells et al., 2013; Fam et al., 2020), and one study was conducted using a late-life depression sample (Li et al., 2022).

Li et al. (2022) examined the effects of a MM intervention for older adults (mean age of 67) with late-life depression (mean age of onset 62) receiving pharmacotherapy (see Table 3). Relative to the control group, the MM group was associated with increased rsFC between: (1) right middle frontal gyrus and right amygdala, (2) right amygdala and right frontal pole, and (3) left supraoccipital lateral cortex and left cerebellum VII. Additionally, decreases in rsFC were seen in: (1) left insula to left precentral gyrus, (2) left insula to right cerebellum VII, and (3) left hippocampus to right intracalcarine cortex. Within the MM group, symptom improvement correlated with amount of time practicing MM (depressive symptoms negatively correlated with minutes of practice), and rsFC between the right middle frontal gyrus and right amygdala (improved anxiety and depression). Lastly, the authors also conducted structural MR analyses which revealed increased white matter integrity (fractional anisotropy) between the medial PFC and amygdala in the MM group at follow up as well.

**TABLE 3:** Prolonged effects of MM on resting-state FC in older adults with cognitive or behavioral changes.

References	Age range	Sample	Demographics	Group by time interaction^A^	Regions	Behavioral correlates/ Additional structural MR findings in MM.
Li et al., 2022	Over 60^RR^	LLD	MM: *N* = 30 (24f, 6 m) Age = 67.66 (5.93) Edu: 13.73 (2.66) TAU: *N* = 30 (22f, 8 m) Age = 67.22 (5.78) Edu: 12.5 (3.08) No information for SES, race, or ethnicity.	MM > TAU MM < TAU	R mfg-R amygdala R amygdala-R frontal pole L supraoccipital lateral cortex-L cerebellum VII L insula-L precentral gyrus, L insula-R cerebellum VII, L hipp-R intracalcarine cortex	Minutes MM practice correlated with lower depression symptoms. Changes in R amygdala-R mfg correlated with improved depression/ anxiety. mfg-amygdala white matter integrity increased in MM group.
Wells et al., 2013	55–90^RR^	MCI	*N* = 14 (13 had both scans) MM: Age = 73 (8) AC: Age = 75 (7) No information provided for sex, edu, SES, race, or ethnicity.	MM > AC	pcc- B mPFC pcc-L hipp	Trend for less bilateral hippocampal volume atrophy at follow up in MM relative to AC.
**Graph metrics**						
Fam et al., 2020	60–85^RR^	MCI	MM: *N* = 19 (12f, 7 m) Age = 72.58 (5.24) Edu: 4.58 (5.19) 18 C, 1 I AC: *N* = 17 (14f, 3 m) Age = 70.71 (6.00) Edu: 3.65 (4.73) 17 C	MM > AC (global eff) MM > AC (nodal eff)	Whole Brain R insula, R anterior medial, and posterior cingulate gyrus, and L stg	

The values within the Age Range column reflect the age range of the study sample in years. Studies that provided only a recruitment age range are provided with the RR superscript. Demographic information and sample sizes reflect sample scanned unless otherwise noted (e.g., in some instances demographic information was available for larger sample but not subset that underwent functional MR scan). Within the Demographics column, mean years of education and age are provided with standard deviation in parentheses. A: This column is included for studies in which an ANOVA was performed and demonstrated a significant treatment-by-time interaction. MM > AC (or MM > TAU) reflects increased FC (or increase in graph metrics) in MM group compared to AC group (or TAU group) after the intervention. MM < TAU reflects less activation or reduced FC in MM group compared to the TAU group after the intervention. A dash indicates a fc between two brain regions. Hipp, hippocampus; mPFC, medial prefrontal cortex; MFG, middle frontal gyrus; PCC, posterior cingulate cortex; STG, superior temporal gyrus; AC, active control; B, bilateral; C, Chinese; Edu, education; F, female; FC, functional connectivity; I, Indian; LLD, late life depression; L, left; M, male; MCI, mild cognitive impairment; MM, mindfulness meditation; N.S., not significant; ROI, region of interest; RSFC, resting-state functional connectivity; R, right, SES, socioeconomic status; N, sample size; TAU, treatment as usual.

Two studies examined rsFC changes following MM in individuals diagnosed with Mild Cognitive Impairment (MCI) (Wells et al., 2013; Fam et al., 2020; see Table 3). Wells et al. (2013) examined MM in older adults (mean ages 73 and 75) diagnosed with MCI compared to treatment as usual (TAU). MM was associated with increased rsFC between (1) bilateral medial prefrontal cortex and posterior cingulate cortex; and (2) left hippocampus and posterior cingulate cortex. A second study examined MM to a psychoeducation control group in older adults (mean ages 71 and 72) with MCI diagnosis (Fam et al., 2020). The authors employed graph metrics to examine topology of resting-state networks and reported at follow-up, the MM group demonstrated preserved temporal global efficiency relative to the control group, which demonstrated a decline in global temporal efficiency. More specifically, the group difference appeared to be driven by maintained nodal efficiency in the MM group (with declines in the control group) in the right insula (salience network), right cingulate (anterior, middle, and posterior cingulate), and left superior temporal gyrus. Further, at follow-up visit, there was an improvement in recognition memory in the MM group as well, with no improvement in control group. See Table 3 for rsFC results from older adult clinical samples.

##### 3.1.3.1. Summary

Within the resting-state literature, 4 articles examined changes in healthy older adults, 2 articles examined changes in older adults with MCI, 1 article examined changes in older adults with late life depression, and 1 article examined changes in middle and older adults diagnosed with breast cancer. Based on two ASL studies and 6 network analyses: MM was associated with increased resting CBF as measured via ASL (in regions of the prefrontal cortex, DMN, and subcortical regions) and was associated with increased rsFC, particularly, between regions of the DMN and PFC. Increased rsFC *within* the DMN was associated with improved cognition, while increased connectivity between amygdala and PFC was linked to better mood. While these studies did not include task MR, the findings point to possible benefits for cognition and emotion regulation based on symptom report and based on clinical samples selected. The following task MR studies will be organized in a similar manner.

### 3.2. Prolonged effects of MM from task fMRI studies: supporting results from younger and middle-age adults during cognitive or emotion tasks

A total of 18 studies were identified that included task functional MR as an outcome measure. Seven studies used a task that included a cognitive component, while the remaining 11 studies examined passive processing of emotionally-salient stimuli (distress, reward, negative emotions, etc.). Regarding methodology, 12 studies utilized a controlled trial design, with control conditions including treatment as usual, waitlist control, and a range of active control conditions. Six studies used a single group, within-subject design. The reader is referred to Tables 4, 5 (and Supplementary Table 1) for information about each of the task fMRI studies discussed in the following section, as well as available information about the sample and intervention used. All but one of the task fMRI studies examined changes in the BOLD signal (one examined changes in ASL). Nearly all studies (17 of 18) reported on sex of the sample. Five of the 18 articles reported data on the sample’s race (2 of which reported limited/incomplete information on ethnicity) with 1 additional study reporting only on the sample’s ethnicity. Six of the 18 reported on educational attainment, and 1 article reported on SES of the sample.

**TABLE 4:** Prolonged effects of MM on activation during cognitive tasks in younger and middle age adults.

References	Age range	Demographics	Task	Within-group MM effect^A^	Group by time^B^	Regions^C^	Behavioral correlates with MM
Huang et al., 2018	25–66	*N* = 23 (21f, 2 m) Age = 48.35 (11.14) All native Mandarin Speakers. No information for edu, race, ethnicity, or SES.	Stroop	Pre < Post deactivation Pre > Post activation		acc, pcc/precuneus (incongruent) R precuneus/cuneus (incongruent)	Pre > Post RT (incongruent)
Carroll et al., 2021	22–69	MM: *N* = 42 (35f, 7 m) Age = 47.17 (12) 22 with fMRI AC: *N* = 41 (38f, 3 m) Age: 43.44 (11.12) 25 with fMRI No information for edu, SES, race, or ethnicity.	eStroop	Pre > Post	NS	B ifg, L mfg, L cingulate/pcc, L claustrum, L itg, L amygdala, L cerebellum, R insular, R hipp/parahipp (general negative)	
Hatchard et al., 2021	37–81	MM (*n* = 11) Age = 48.36 (11.37) WL (*n* = 10) Age = 56.5 (8.11) 90% W No information for sex, edu, SES, or ethnicity.	eStroop	Pre > Post	NS	L mfg, L ifg, L precuneus, L postcentral gyrus, and L calcarine gyrus (affective-neutral)	Slower RT for affective-neutral contrast at follow up for MM but not AC
Allen et al., 2012	18–50^RR^	MM: *N* = 19 (11f, 8 m) Age = 27 AC: *N* = 19 (10f, 9 m) Age = 26 No information for edu, SES, race, or ethnicity.	eStroop	MM Dose	MM > AC	L DLPFC/rostral pfc (congruent + incongruent > passive) R anterior insula, L sfg, acc, dorsal cc, B superior medial frontal (negative)	↓ Stroop Conflict RT MM dose positively correlated with improved response inhibition accuracy on different task.
Tomasino et al., 2016	21–35	*N* = 16 (12f, 4 m) Age = 30.2 (4.5) Edu: 15 (2.23) No information for SES, race, or ethnicity.	LM vs. LT	Pre < Post		B Mid Orbital Gyrus	Faster RTs. R mid-orbital activation correlated personality change
Bachmann et al., 2018	18–65	MM: *N* = 21 (13f, 8 m) Age = 40 (10.58) AC: *N* = 19 (9f, 10 m) Age = 40.26 (13.81) No information for edu, SES, race, or ethnicity.	N-Back	Pre < Post^MM^	NS	B inferior parietal, R posterior insula R precuneus (Correct-Non-target on 1-back)	Cognitive symptom improvement correlated with L putamen, globus pallidus, and thalamus. Behavioral symptom improvement correlated with mPFC and PCC.
Seminowicz et al., 2020	18–65^RR^	*N* = 98 (89f, 9 m) Age = 36 71 W; 17 AA; 9 O Some college: 20 College: 78 No information for ethnicity or SES.	MSIT		MM < AC	B Cuneus and R Parietal operculum (difficult vs. easy)	

The values within the Age Range column reflect the age range of the study sample in years. Studies that provided only a recruitment age range are indicated with the RR superscript. Demographic information and sample sizes reflect sample scanned unless otherwise noted (e.g., in some instances demographic information was available for larger sample but not subset that underwent functional MR scan). Within the Demographics column, mean years of education and age are provided with standard deviation in parentheses when data was available. For one study, education was provided with categorical descriptors and values reflect sample sizes. When race was available, values in table correspond to sample size. One study conducted outside of the US provided only language spoken (that variable was included within Demographics column). A: This column is included for studies that demonstrate a significant effect of time within the MM group. Pre < Post reflects a within-group contrast with greater activation (or deactivation when noted) at the follow up visit (after MM intervention) compared to baseline scan. Pre > Post reflects a within-group contrast with reduced activation (or deactivation when noted) at the follow up visit compared to baseline scan. B: This column is included for studies in which an ANOVA was performed and demonstrated a significant treatment-by-time interaction (treatment is MM or AC and time is pre or posttreatment). MM > AC reflects greater activation in MM compared to AC after the intervention. MM < AC contrast reflects less activation in MM compared to AC after the intervention. C: This column includes corresponding significant regions of the brain associated with contrasts in A or B. When appropriate, the specific task contrast is included in parentheses as well. ACC, anterior cingulate cortex; DLPFC, dorsolateral prefrontal cortex; Hipp, hippocampus; IFG, inferior frontal gyrus; MFG, middle frontal gyrus; mPFC, medial prefrontal cortex; MTG, middle temporal gyrus; parahipp, parahippocampus; PCC, posterior cingulate cortex; PFC, prefrontal cortex; SFG, superior frontal gyrus; STG, superior temporal gyrus; AC, active control; AA, African American; A, Asian; B, bilateral; Edu, education; eStroop, emotional Stroop; F, female; HC, healthy adult; L, left; M, male; MM, mindfulness meditation; MSIT, Multi-Source Interference Task; LM vs. LT, Little Man Versus Letter Task; N, sample size; NS, not significant; O, Other; RT, reaction time; R, right; SES, socioeconomic status; W, white.

**TABLE 5:** Prolonged effects of MM on activation or FC during emotion tasks in younger and middle age healthy adults.

References	Age range	Demographics	Task	Within-group MM effect^A^	Group by time^B^	Regions^C^
Kral et al., 2018	26–66	MM: *N* = 32 (22f, 10 m) Age = 50.8 (8.8) AC: *N* = 35 (19f, 16 m) Age = 48.1 (12.2) No information for edu, SES, race, or ethnicity.	Emotion Processing		MM < AC MM > AC	R amygdala (positive > neutral) R amygdala-vmPFC (negative-neutral, positive-neutral)
Desbordes et al., 2012	22–55^RR^	MM: *N* = 12 (8f, 4 m) Age = 34.3 (9.6) AC1: *N* = 12 (9f, 3 m) Age = 32.0 (5.4) AC2: *N* = 12 (5f, 7 m) Age = 36.0 (7.6) No information for edu, SES, race, or ethnicity.	Emotion Processing	Pre > Post	MM < AC2	R amygdala (positive images) R amygdala (across all valences)
Kirk et al., 2014	–	MM: *N* = 26 (14f,12 m) Age = 32.2 (10.4) AC: *N* = 26 (15f,11 m) Age = 31.3 (10.1) MM: *N* = 17 (10f,7 m) Age = 32.7 (11.1) AC: *N* = 17 (9f, 8 m) Age = 32.4 (11.4) No information for edu, SES, race, or ethnicity.	Reward Processing		MM > AC MM < AC	L mid/anterior insula vmPFC Negative FC between insula and vmPFC in MM group.
Leung et al., 2018	25–55^RR^	MM: *N* = 10 (5f, 5 m) Age = 37.8 (11.2) Edu = 15.6 (5.4) 10 C AC: *N* = 10 (6f, 4 m) Age = 42.4 (8.5) Edu = 19.8 (3.6) 10 C	Emotion Processing		MM < AC	R amygdala (negative and neutral conditions) R amygdala (negative stimuli) correlated with MM practice

The dash within the Age Range column indicates the age range of the study sample was not provided in the manuscript. The values within the Age Range column reflect the age range of the study sample in years. Studies that provided only a recruitment age range are provided with the RR superscript. Demographic information and sample sizes reflect sample scanned unless otherwise noted (e.g., in some instances demographic information was available for larger sample but not subset that underwent functional MR scan). Within the Demographics column, mean years of education and age are provided with standard deviation in parentheses when data was available. When ethnicity was available, values in table correspond to sample size. A: This column is included for studies that demonstrate a significant effect of time within the MM group. Pre < Post reflects a within-group contrast with greater activation or increased FC at the follow up visit (after MM intervention) compared to baseline scan. Pre > Post reflects a within-group contrast with reduced activation or decreased FC at the follow up visit compared to baseline. B: This column is included for studies in which an ANOVA was performed and demonstrated a significant treatment-by-time interaction (treatment is MM or AC and time is pre or posttreatment). MM > AC reflects greater activation or increased FC in MM compared to AC after the intervention. C: This column includes corresponding significant regions of the brain associated with contrasts in A or B. When appropriate, the specific task contrast is included in parentheses. A dash is used to indicate fc between two brain regions. vmPFC, ventromedial prefrontal cortex; AC, active control; B, bilateral; C, Chinese; Edu, education; F, female; HC, healthy adult; L, left; M, male; MM, mindfulness meditation; N, sample size; R, right; SES, socioeconomic status.

#### 3.2.1. MM and cognitive functional MR tasks in younger and middle-age adults

Seven task fMRI studies were identified that examined changes in the BOLD signal during performance of a cognitive task (see Table 4). Two studies were conducted with healthy adults, four studies were conducted with subclinical samples (pain, bereavement, high stress), and one study was conducted with a clinical sample (attention deficit/hyperactivity disorder). Across studies, the tasks examined involved attention (working memory, sustained attention) or executive function (response inhibition, perspective-taking, and decision-making). Three of the studies examined cognition with an emotion component.

Two studies conducted within healthy adults both reported *increased* prefrontal activation and improved task performance during an attention/executive functioning task (Allen et al., 2012; Tomasino et al., 2016). More specifically, MM was associated with faster reaction time and greater bilateral PFC activation (orbitofrontal cortex) during a perspective taking task (Tomasino et al., 2016). Similarly, increased activation in the left dorsolateral and rostral PFC and faster reaction time was observed during the emotional Stroop task (Allen et al., 2012). In that study, dose of MM was examined as well and total number of minutes practicing MM was associated with both better task performance and increased recruitment of prefrontal regions (including dorsal anterior cingulate cortex (ACC), medial PFC, superior frontal gyrus) and right anterior insula during the response inhibition task (Allen et al., 2012).

Four studies examined changes in subclinical samples and reported *decreased* activation during an attention/executive function task following MM (with either equivalent or improved performance). Huang et al. (2018), examined response inhibition within a bereavement sample and reported decreased activation in the precuneus/cuneus, increased *deactivation* in the ACC and PCC/precuneus and faster reaction times for incongruent trials after MM (Huang et al., 2018). Three studies reported *decreased* activation in the context of equivalent performance. Seminowicz et al. (2020) examined response inhibition in adults with migraines. In that study, decreased activation was observed in the bilateral cuneus and right parietal operculum during the more difficult trials despite equivalent task performance (Seminowicz et al., 2020). Processing negative stimuli on the emotional Stroop, MM was associated with decreased activation in regions of the DMN (inferior frontal gyrus, cingulate/PCC, inferior temporal gyrus, hippocampus/parahippocampus) and emotion-generating regions (amygdala, claustrum) among highly stressed teachers with equivalent task performance (though group by time interaction did not reach significance) (Carroll et al., 2021). Similarly, among breast cancer survivors with chronic neuropathic pain, decreased activation in left middle frontal, inferior frontal, precuneus, postcentral gyrus and calcarine gyrus when processing affective relative to neutral stimuli on emotional Stroop (with equivalent performance) compared to baseline (group by time interaction did not reach significance) (Hatchard et al., 2021).

Finally, one study examined attention/executive function in a clinical sample (Bachmann et al., 2018). Bachmann et al. (2018) examined working memory changes in adults diagnosed with attention deficit hyperactivity disorder. In that study, MM was associated with increased activation in DMN hubs (bilateral inferior parietal lobule, right precuneus) and insula (right posterior insula) (compared to baseline) during a working memory task (n-back) (Bachmann et al., 2018). While behaviorally, there were no differences between groups in accuracy or reaction time during the task, bilateral medial prefrontal cortex (PFC) and bilateral posterior cingulate cortex (PCC) activation during the task was significantly correlated with improvement in behavioral symptoms (hyperactivity and restlessness).

##### 3.2.1.1. Summary

Across the seven studies that used functional task-based MR to examine the effects of MM on functional activation during an attention/executive function task, within healthy younger and middle-age adults, *increased* activation within the PFC was linked to improved performance. In subclinical samples, MM was linked to *decreased* activation in PFC which correlated with equal or better performance. However, in a clinical sample with a baseline weakness in attention/executive function, *increased* activation during the task was linked to improved behavioral symptoms but not task performance. Overall, not unexpectedly, the findings in clinical and subclinical samples were more mixed: but in general highlighted the role of the PFC as well as the default mode network.

#### 3.2.2. MM and emotion functional MR tasks in younger and middle-age adults

Eleven task-based MR studies were identified that examined passive processing of an emotionally salient experience. Four studies were conducted in healthy adults (see Table 5), four were conducted in a subclinical (pain or elevated stress) sample (see Table 6), and three were conducted in a clinical (prior psychiatric diagnosis) sample (see Table 7). The specific task employed varied: five studies focused on emotion processing (emotionally valent stimuli or induction of emotion state), two studies focused on the stress response, one study examined reward response, and three studies used self-referential statements linked to psychopathological symptoms.

**TABLE 6:** Prolonged effects of MM on activation during emotion tasks in younger and middle age adults reporting elevated stress.

References	Demographics	Task	Within-group MM effect^A^	Group by time^B^	Regions^C^	Behavioral correlate with MM
Braden et al., 2016	MM: *N* = 12 (8f,4 m) Age = 46 (11.3) Edu = 14.5 (2.5) AC: *N* = 11 (6f, 5 m) Age = 43 (2.5) Edu = 14.5 (2.8) No information for SES, race, or ethnicity.	Sadness Induction	Pre < Post	NS	L sgACC, R sgACC, L vlPFC (sadness induction)	Sadness intensity rating for task positively correlated with L sgACC.
Monti et al., 2012	MM: *N* = 8 (8f) AC: *N* = 10 (10f) Full sample: 5 AA; 1 A; 12 W No information for ethnicity, edu, or SES.	Stress Task	Pre > Post		PCC (stressor condition)	
Turpyn et al., 2021	*N* = 20 (20f,0 m) Age = 48.5 (7.62) > $100k 60%; $75–100k: 5% $60–74.9k: 5% $45–59.9k: 10% < $45k: 15% No information for edu, race, or ethnicity.	Emotion Processing		MM > AC	L posterior insula (negative > neutral)	Lower cortisol reactivity and observer rating of treatment response (shared positive emotion between child and parent during stressful interaction) positively correlated with posterior insula.
Farb et al., 2010	MM: *N* = 20 (12f, 4 m) Age = 42 WL: *N* = 16 (15f, 5 m) Age = 45.55 No information for edu, SES, race, or ethnicity.	Sadness Provocation		MM < AC deactivation MM < AC activation	R BG, R insula, R sgACC, vmPFC, R vlPFC, R sfg (sad vs. neutral) R precuneus/pcc, L PFC, frontal operculum, superior temporal sulcus, and itg (sad vs. neutral)	Mood symptoms negatively correlated with R insula/R lPFC deactivation and positively correlated with L superior temporal sulcus activation.

Demographic information and sample sizes reflect sample scanned unless otherwise noted (e.g., in some instances demographic information was available for larger sample but not subset that underwent functional MR scan). Within the Demographics column, mean years of education and age are provided with standard deviation in parentheses when data was available. When ethnicity and/or race was available, values in table correspond to sample size. One study provided categorical descriptors for SES and for that study percentage values are noted reflecting percent of sample in each bracket. A: This column is included for studies that demonstrate a significant effect of time within the MM group. Pre < Post reflects a within-group contrast with greater activation at the follow up visit (after MM intervention) compared to baseline. Pre > Post reflects a within-group contrast with reduced activation at the follow up visit compared to baseline. B: This column is included for studies in which an ANOVA was performed and demonstrated a significant treatment-by-time interaction (treatment is MM or AC and time is pre or posttreatment). MM > AC reflects greater activation (or deactivation if noted) in MM compared to AC after the intervention. MM < AC contrast reflects less activation (or deactivation if noted) in MM compared to the AC after the intervention. NS reflects instances where a group time interaction was examined but was not statistically significant (in which case no regions will be listed in the regions column). C: This column includes corresponding significant regions of the brain associated with contrasts in A or B. When appropriate, the specific task contrast is included in parentheses. BG, basal ganglia; ITG, inferior temporal gyrus; IPFC, lateral prefrontal cortex; PCC, posterior cingulate cortex; PFC, prefrontal cortex; sgACC, subgenual anterior cingulate cortex; SFG, superior frontal gyrus; vmPFC, ventromedial prefrontal cortex; vlPFC, ventrolateral prefrontal cortex; AC, active control; BDI, Beck Depression Index; B, bilateral; Edu, education; F, female; HC, healthy adult; IAPS, international affective picture system; L, left; M, male; MM, mindfulness meditation; N, sample size; N.S., not significant; R, right; SES, socioeconomic status; WL, waitlist.

**TABLE 7:** Prolonged effects of MM on activation during emotion tasks in younger and middle age adults with prior psychiatric diagnosis.

References	Demographics	Within-group MM effect^A^	Group by time^B^	Regions^C^	Behavioral correlate with MM
Goldin and Gross, 2010	*N* = 16 (9f, 7 m) Age = 35.2 (11.9) Edu 16.3 (3.5) 5 A; 2 LA; 1 NA 8 W 14 (8f,6 m) with fMRI No information for SES.	Pre < Post Pre > Post		Inferior/superior parietal lobule, cuneus, precuneus, middle occipital gyrus, parahipp (breath-focused attention vs. react negative) R amygdala (react negative)	Symptom reduction correlated with medial cuneus, L cuneus, and R middle occipital gyrus (breath-focused attention versus react negative) Reduced self-reported negative emotion to cues when breath-focused strategy used.
Goldin et al., 2012	MM: *N* = 31 (19f, 12 m) Age = 32.87 (8.83) Edu = 16.4 (2) 14 A; 3 H; 1 Multir; 13 W AC: *N* = 25 (10f, 15 m) Age = 32.99 (7.97) Edu = 16.84 (2.64) 11 A; 1 H, 3 Multir 10 W 42 with fMRI scans.		MM > AC	pcc (Negative vs. Case) Differences in timing of neural responses during negative versus case were seen in dmPFC and pcc after intervention.	Symptom improvement correlated with dmPFC (positive correlation for negative versus case; negative correlation for positive versus case.
Williams et al., 2020	*N* = 16 (13f, 3 m) Age = 34.6 (9.4) No information for edu, SES, race, or ethnicity.	Pre > Post		B dACC/medial superior frontal gyrus (self-blame > other-blame) L precentral and L postcentral gyrus (self-blame > -fixation)	Symptoms improvement correlated with change in pcc/precuneus during task (self-blame > fixation)

Demographic information and sample sizes reflect sample scanned unless otherwise noted (e.g., in some instances demographic information was available for larger sample but not subset that underwent functional MR scan). Within the Demographics column, mean years of education and age are provided with standard deviation in parentheses when data was available. When race or ethnicity was available, values in table correspond to sample size (or percentage of sample, indicated with% sign). A: This column is included for studies that demonstrate a significant effect of time within the MM group. Pre < Post reflects a within-group contrast with greater activation or FC at follow up (after MM intervention) compared to baseline. Pre > Post reflects a within-group contrast with reduced activation or FC at the follow up visit (after MM intervention) compared to baseline scan (before MM intervention). B: This column is included for studies in which an ANOVA was performed and demonstrated a significant treatment-by-time interaction (treatment is MM or AC and time is pre or posttreatment). MM > AC reflects greater activation or increased FC in MM compared to AC after the intervention. C: This column includes corresponding significant regions of the brain associated with contrasts in A or B. When appropriate, the specific task contrast is included in parentheses. BG, basal ganglia; dACC, dorsal anterior cingulate cortex; dmPFC, dorsomedial prefrontal cortex; parahipp, parahippocampus; PCC, posterior cingulate cortex; PFC, prefrontal cortex; sgACC, subgenual anterior cingulate cortex; vlPFC, ventrolateral prefrontal cortex; AC, active control; AA, African American; A, Asian; B, bilateral; BC, diagnosed with breast cancer; Edu, education; F, female; HC, healthy adult; H, Hispanic; L, left; M, male; MM, mindfulness meditation; Multir, multiracial; NHW, non-Hispanic White; N, sample size; N.S., not significant; O/NR, other or not reported; R, right; SES, socioeconomic status; W, white.

Three studies examined response to images with emotional valence (positive or negative) in healthy adults and reported decreased activation in the amygdala relative to a control group (Desbordes et al., 2012; Kral et al., 2018; Leung et al., 2018). More specifically, the first study reported decreased activity in amygdala for positive relative to neutral stimuli (no difference for negative relative to neutral) and increased functional connectivity between amygdala and prefrontal cortex during processing of positive or negative emotional stimuli (relative to neutral stimuli) (Kral et al., 2018) at follow up. A second study also reported decreased amygdala activation for positive stimuli, as well as decreased amygdala activation for stimuli collapsed across all valences (no specific effect for negative stimuli) (Desbordes et al., 2012) following MM. Finally, a third study reported decreased amygdala activity for both neutral and negative stimuli (Leung et al., 2018). In that study, a dose-response effect was demonstrated as well (amount of time practicing MM significantly correlated with decreased amygdala activation when viewing negative stimuli) (Leung et al., 2018). A fourth study in healthy adults during reward processing found MM (compared to AC group) was associated with increased insula and decreased ventromedial PFC activation (Kirk et al., 2014).

Two studies used a sadness induction task in a subclinical sample (pain, stressed) and reported changes in PFC activation (among other findings). More specifically, Braden et al. (2016), reported within a sample of middle-aged adults (mean age 43 and 46) with chronic pain there was increased activation in prefrontal regions (subgenual anterior cingulate cortex and ventrolateral PFC; with trends in dorsomedial PFC and right ventrolateral PFC) in the MM group during sadness condition (though the group by time interaction did not reach significance) (Braden et al., 2016). Similarly, Farb et al. (2010) examined the effects of MM within a sample of middle-aged adults (mean age of 46) with elevated stress and found less *deactivation* in the PFC (including subgenual ACC, ventromedial PFC, ventrolateral PFC, superior frontal gyrus) and insula and less activation (precuneus/PCC, PFC, inferior temporal gyrus) following MM during sadness induction relative to the control group. Further, deactivation in insula and lateral PFC during that task correlated with improvements in depression (greater deactivation correlated with better mood in MM) (Farb et al., 2010).

Two studies attempted to induce a stress response. Monti et al. (2012) examined changes in cerebral blood flow (as measured using ASL) among older adult women with history of cancer (52–77 years of age), and found decreased cerebral blood flow perfusion within the posterior cingulate cortex during a stressful cognitive task (Monti et al., 2012). One study examined the relationship between processing of stressful stimuli and cortisol within a subclinical sample. More specifically, Turpyn et al. (2021), conducted a study with mothers of adolescents that reported significant stress related to their child’s behavior (mean age 48.5 years old). Greater insula activation during stressor task (viewing negative versus neutral stimuli) was observed after MM intervention (Turpyn et al., 2021). Further, insula activation during that task was correlated with: a reduction in cortisol reactivity during a different stress task as well as task performance improvements on a stress task based on observer ratings (Turpyn et al., 2021). See Table 6 for sadness induction and stress studies.

Three studies conducted with psychiatric samples had participants process negative self-referential statements linked to underlying psychopathology (See Table 7). Among individuals in remission from major depressive disorder, when viewing negative self-referential statements (self-blame statements), activation was reduced in the dorsal ACC/medial superior frontal gyrus (self-blame versus other-blame) and left precentral and postcentral gyrus) (self-blame versus rest condition) after MM (Williams et al., 2020). Within that study, reduced activation in PCC/precuneus correlated with symptom improvement. Two studies examined responses to negative self-referential statements in individuals with an anxiety disorder. Among individuals diagnosed with social anxiety disorder, decreased amygdala activation was observed following MM when processing negative emotional stimuli (Goldin and Gross, 2010). Additionally, when asked to utilize a previously learned MM strategy to process negative stimuli, participants reported less negative emotions during those trials and additional activation was observed in multiple regions of the DMN (inferior and superior parietal lobule, precuneus, parahippocampal gyrus). Similarly, a second study of social anxiety disorder, reported increased PCC when viewing negative stimuli (negative self-referential statements) following MM compared to AC (Goldin et al., 2012). Finally, within the MM group, greater dorsomedial PFC activation during the task at follow up was associated with symptom improvement as well as changes in timing of dorsomedial PFC and PCC task activation (Goldin et al., 2012).

##### 3.2.2.1. Summary

Across the five articles that examined emotion processing (either viewing of emotion cues or induction of sadness), following MM, there was a shift in how emotions were processed either via decreased activation in emotion-generating regions (e.g., amygdala) or via increased activation in emotion-regulatory processes (e.g., increased prefrontal cortex/insula activity, or increased connectivity between prefrontal cortex and amygdala). MM impacted processing of stress as well, potentially via differential engagement in DMN (decreased posterior DMN in two articles), as well as some suggestion for increased recruitment of regulatory regions, though this varied by population (increased insula activation in stressed mothers which correlated with cortisol). Similarly, in the context of psychopathology, there was decreased posterior activation (albeit precentral and postcentral gyrus) as well as increased activity in regulatory prefrontal regions (dorsal ACC/medial superior frontal). In social anxiety, decreased amygdala activity was observed after MM when viewing cues, with increased DMN activation when using MM-based strategy.

## 4. Discussion

**FIGURE 1:**
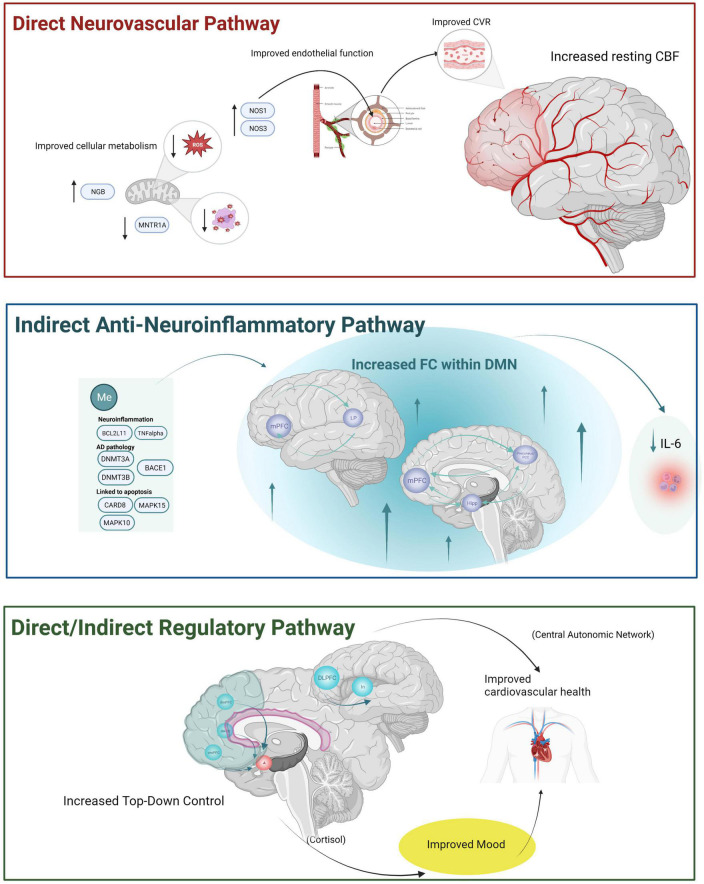
The three hypothesized neurovascular pathways by which MM may have beneficial effects for older adults based upon the review of the literature. A direct neurovascular mechanism is depicted at the top of the figure within the red box reflecting increased resting-state cerebral blood flow observed using ASL among cognitively unimpaired older adult’s samples. This mechanism is thought to reflect improved NVU function via improvements in (1) cellular metabolism (e.g., decreased reactive oxidative stress and free radicals via MM induced DNA methylation like increased NGB, decreased MNTR1A resulting in improved cellular metabolism within mitochondria) and improved endothelial function via increased NOS1 and NOS3. Improvements in NVU then resulting in improved CVR and increased resting CBF. An anti-neuroinflammatory mechanism is depicted within the middle blue box and is hypothesized to be an indirect neurovascular pathway reflecting increased resting state functional connectivity within the default mode network observed in both cognitively unimpaired older adults and older adults with cognitive or behavioral changes. Decreases in neuroinflammatory processes are conceptualized as an indirect neurovascular mechanism (with downstream effects on NVU not depicted here). We hypothesized this mechanism may reflect anti-inflammatory processes relevant for both aging as well as AD-specific pathology. A final regulatory-control mechanism is depicted within the bottom green box and is thought to reflect both a direct and an indirect neurovascular pathway based upon (1) increased involvement of PFC in older adults after MM and based on (2) increased PFC involvement during task performance in younger and older adult’s samples. Increased PFC (along with decreased activation of emotion generating regions during emotion tasks) may reflect improved mood and reduced stress, both of which can have an indirect positive benefit on neurovascular health. A more direct mechanism via increased FC within the central autonomic network (responsible for controlling cardiovascular function more directly) would reflect a more direct pathway.BACE1, beta-secretase 1; BCL2L11, BCL2 like 11; CARD8, caspase recruitment domain family member 8; CBF, cerebral blood flow; CVR, cerebrovascular reactivity; DACC, dorsal anterior cingulate cortex; DLPFC, dorsolateral prefrontal cortex; dmPFC, dorsomedial prefrontal cortex; DMN, default mode network; DNMT3A, DNA methyltransferase 3 alpha; DNMT3B, DNA methyltransferase 3 beta; FC, functional connectivity; hipp, hippocampus; IL-6, interleukin 6; In, insula; LP, lateral parietal; MAPK15, mitogen-activated protein kinase 15; MAPK10, mitogen-activated protein kinase 10; MNTR1A, melatonin receptor type 1A gene; Me, methylation; mPFC, medial prefrontal cortex; NOS1, nitric oxide synthase 3, NOS3, nitric oxide synthase 3; NGB, neuroglobin; PCC, posterior cingulate cortex; ROS, reactive oxidative stress; vmPFC, ventromedial prefrontal cortex, TNFalpha, tumor necrosis factor alpha. Created with BioRender.com.

Neurovascular processes are integral to an understanding of typical and pathological brain aging (Tarantini et al., 2017; Abdelkarim et al., 2019; Korte et al., 2020; Nielsen et al., 2020; Tsvetanov et al., 2021). We reviewed experimental MM studies that included functional MR, an estimate of neurovascular function, as an outcome measure. While there is a growing body of literature demonstrating functional MR changes following MM in younger and middle-aged adults, there is a much smaller set of studies examining these effects exclusively within an older adult sample. We identified 7 articles that examined resting-state MR as an outcome measure in an older adult sample. While none of the articles included task-based MR, findings from these studies suggest functional brain changes in older adults may be associated with cognitive or emotional processing improvements. Findings from exclusively older adult samples were reviewed first followed by review of findings from cognitive and emotion processing task MR studies across a wider population (younger and middle age adults, clinical and healthy adults). Based on these studies, we propose three potential neurovascular pathways by which MM may benefit older adults (see Figure 1). Each mechanism will be briefly summarized and then discussed within the broader context of normal and pathological aging literature.

### 4.1. Increased resting CBF: a potential direct neurovascular pathway

Two studies examined changes in CBF as measured using ASL in older adults and both studies reported resting state CBF increased following a MM intervention (particularly within DMN and PFC). Reduced CBF perfusion is observed in healthy older adults (Du et al., 2017) and has been linked to cognitive decline in older adults (Viticchi et al., 2012; Vemuri et al., 2015; Takeda et al., 2020). Moreover, reduced CBF is thought to reflect a mechanism driving cognitive decline and potentially neurodegeneration, in part via worsened neurovascular health (e.g., worsened cerebrovascular reactivity) stemming from NVU disruption. These preliminary findings reported in older adults provide initial support that MM has the potential to improve a key marker of neurovascular function in older adults: resting state CBF. While increased resting state CBF might suggest an improvement in NVU function, this has not been directly assessed following MM. Interestingly, work in adults with glaucoma, has provided some initial links between MM, the NVU and brain function in a relatively older sample (Gagrani et al., 2018). Using functional near-infrared spectroscopy, the authors demonstrated a link between increased activation in the PFC following MM was linked to reduced reactive oxidative stress and improved vascular health; potentially via changes in DNA methylation of genes involved in endothelial function, cellular metabolism and nitric oxide synthetase (Dada et al., 2018, 2021a,b). Further support for the potential of MM to impact processes of cellular metabolism relevant for CBF may be gleaned from acute studies of MM. Using phosphorous magnetic resonance spectroscopy (^31^ P-MRS), for instance, Galijasevic et al. (2021) reported changes in multiple cerebral metabolites that play a key role in cellular metabolism during MM practice (e.g., altered intracellular pH, changes in level of metabolites such as Pi, as well as changes in ratios of select metabolites that would suggest changing ATP turnover) (Galijasevic et al., 2021).

### 4.2. Increased FC within the DMN: a potential indirect anti-neuroinflammatory pathway

Seven studies examined changes in functional connectivity in older adults. Despite notable heterogeneity in methods and analyses employed, all studies reported *increased* functional connectivity following MM and generally, all studies reported increases within components of the DMN. Interestingly, 2 of the articles that examined DMN connectivity in older adults reported on cognitive function providing further support for this link. More specifically, in healthy older adults, improved cognitive functions following MM were correlated with increased DMN functional connectivity (especially, hippocampus, precuneus and angular gyrus) (Sevinc et al., 2021), while in older adults with MCI, those that received MM maintained nodal efficiency (with declines in control group) within the right insula, right cingulate and left superior temporal gyrus and showed less cognitive decline at follow up.

The potential for MM to increase functional connectivity within the DMN is of particular interest for Alzheimer’s Disease (AD). Briefly, AD has been described as a synaptopathy, or a “disease of circuits” (Marttinen et al., 2018), with the disrupted circuitry found to contribute to both the pathogenesis of AD and the emergence of clinical symptoms (cognitive decline and behavior changes) (Marttinen et al., 2018). Functional changes in preclinical AD occur initially in the DMN thought to reflect synaptic loss mediated by microglial and astrocyte inflammatory processes and coincide with amyloid buildup (Zhu et al., 2019; Wang et al., 2020). Increasing evidence has emphasized the role of neuroinflammation in AD, and its role in the disruption of brain connectivity (Passamonti et al., 2019) especially within DMN which has motivated the development of anti-inflammatory interventions for AD (Du et al., 2017; Passamonti et al., 2019; Huang et al., 2020). Interestingly, one study in younger/middle-aged adults (see Supplementary Table 2) reported increased rsFC in DMN after MM was correlated with a decrease in a pro-inflammatory marker at follow-up (Creswell et al., 2016) providing a more direct potential link between MM, increased DMN functional connectivity and the NVU via an anti-neuroinflammatory mechanism. While this mechanism has not been studied in older adults, there is evidence MM has a beneficial impact on reducing inflammation in healthy adults (Creswell et al., 2012) and older adults with MCI (Ng et al., 2020), and MM has been linked to changes in DNA methylation related to neuroinflammatory processes and glial activation in which are relevant for AD (Dada et al., 2021a).

### 4.3. Linking brain changes to assessment measures: potential direct and indirect neurovascular pathway via top-down control

Resting-state MR studies in older adults and task-based MR studies in middle and younger aged adults provide support for increased activation within PFC regions following MM. Prior reviews have suggested this increase in PFC engagement may reflect the potential of MM to strengthen compensatory mechanisms seen in aging (Smart et al., 2021) [i.e., the shift toward greater recruitment of PFC regions with increasing cognitive demands as a mechanism to compensate for age-related neurodegeneration (Sebastian et al., 2013)]. Potentially, increased PFC activation may also signify enhancement of a broader top-down control mechanism that could have downstream effects on neurovascular health via improved stress and emotion regulation. While not examined directly, in older adults with depression, MM was associated with increased FC between amygdala and PFC (Li et al., 2022) and improved mood at follow-up. Further support for enhanced top-down control comes from the task MR studies, which found greater PFC activation and increased FC between PFC to limbic regions during cognitive and emotion regulation tasks, as well as, greater insula activation and reduced amygdala activation in stress and emotion tasks. Finally, strengthening of top-down control (as measured via task fMRI and via functional connectivity studies) may also reflect a more direct neurovascular pathway as well via enhancement of the neural network involved in the regulation of cardiovascular function [e.g., central autonomic network (Beissner et al., 2013)]. Future work exploring this mechanism would be of interest as well.

### 4.4. Future directions

Future research with more rigorous neuroimaging methodology is needed to fully elucidate the proposed neurovascular mechanisms hypothesized in the present review. First, a notable gap in the literature is the absence of task MR studies older adult samples. Task MR is an excellent tool for studying how MM might translate to tasks and activities outside of the practice. Different patterns of activation/deactivation emerge with age on both cognitive and emotion processing tasks (Sebastian et al., 2013; McDonald et al., 2018; Rieck et al., 2021). Future studies should examine the effects of MM using cognitive and emotion processing functional MR tasks in older adult samples and symptom improvement should be examined in relation to fMRI changes. Secondly, future studies should also measure regional changes in BOLD (i.e., regional homogeneity, fractional amplitude of low-frequency fluctuations) in older adults, as measures of local brain function demonstrate lower intraindividual variability relative to global metrics of brain function (Chen et al., 2015; Larabi et al., 2021).

To fully appreciate the impact of MM on neurovascular health in older adults, future studies should examine NVU function more directly via cerebrovascular reactivity (CVR). As mentioned, CVR is a more direct measure of vascular endothelium and smooth muscle function (relative to baseline CBF) that shows predictable changes in the context of normal and pathological aging. Surprisingly, CVR has not been examined directly as a mechanism of change driving MM effects, nor has it been included as a covariate in MM studies examining functional MR changes. Since it has been recommended all studies that purport to examine functional MR in older adults should employ methodology that can properly account for CVR this is a surprising gap in the literature (as functional MR metrics are influenced by age-related changes in CVR) (Tsvetanov et al., 2021). Notably, while early studies of CVR relied on gas inhalation to quantify CVR, recent work has demonstrated CVR can be measured successfully and reliably using a breath holding task (Cohen and Wang, 2019; Cohen et al., 2021), thus eliminating the need for more expensive, higher risk procedures.

Unfortunately, only 8 of the 25 articles reviewed in the main text of this review reported any racial or ethnic characteristics of their sample. Of the 8 studies that did include any information on race or ethnicity, the vast majority of studies were conducted in samples that were primarily White (with few studies reporting on ethnicity of the sample). This is a significant gap in the literature that needs to be addressed. While preliminary evidence suggests the benefits of MM for older adults will have benefits for older adults from minoritized backgrounds (Szanton et al., 2011; Palta et al., 2012), additional research is needed to understand this. Given the significant health disparities in vascular and cognitive health, additional research is needed to disentangle the impact of institutionalized, personally mediated, and internalized racism on neurovascular and cognitive health among older adults from minoritized backgrounds (Williams and Ovbiagele, 2020). Prior work has highlighted the complex interplay between experiences of racism and discrimination, mood and vascular risk factors in older adults (Zahodne et al., 2020), and has highlighted the role of neuroinflammatory and neurovascular processes in understanding cognitive aging in older adults from minoritized backgrounds (Zahodne et al., 2019; Boots et al., 2022; Manning et al., 2022). Additionally, while most studies included information regarding the sex of the participants, this variable was not examined directly which may be of interest to consider further (e.g., interactions with sex may be of interest for emotion regulation strategies). Given the complex interplay between physiological factors, age, and demographic variables future studies using CVR must account for these variables within analyses.

## Author contributions

All authors made substantial contributions to the conception and design of the work, the analysis and interpretation of data for the work, drafting and revising of the manuscript, gave final approval of the version to be published, and agree to be accountable for all aspects of the work.
