# Supplementary material for: Three potential neurovascular pathways driving the benefits of mindfulness meditation for older adults
Source: Front Aging Neurosci. 2023 Jun 29;15:1207012. doi: 10.3389/fnagi.2023.1207012 (PMC10340530; doi:10.3389/fnagi.2023.1207012)
Supplement: Supplementary file 1 [file Table_1.DOCX]

Supplemental Table 1: Additional information on methodology for studies included in Tables 1 through 7.

| **Study** | **Sample** | **MM Intervention** | **MM Dose** | **Control Condition** | **Method** | **Analysis Approach** |
| --- | --- | --- | --- | --- | --- | --- |
| **Arterial Spin Labeling (ASL) Studies in Older Adults.** | | | | | | |
| Moss et al., 2022 | Healthy controls | MBSR | Length: 8 weeks  Frequency: Weekly  Duration: 2 hours  Homework: Recommended daily home practice (25-30 minutes per day) | Within-Subject Design | Approximately 6 minutes  Asymmetric Pulsed ASL Sequence  3T Siemens mMR PET–MRI | Pre vs Post contrast  Whole brain analysis |
| Monti et al., 2012 | Patients with a history of breast cancer | MBAT | Length: 8 weeks  Frequency: Weekly  Duration: 2.5 hours  Homework: Encouraged to practice at home. | Educational Support | 8 min continuous and pseduo-continuous ASL (CASL)  3T Siemens Trio scanner | Group by Time interaction effect: (MBAT: post versus pre) versus (control: post- versus pre)  Also reported within group effect (post versus pre) for MBAT and control group separately.  Whole brain analysis |
| **Resting State Functional Connectivity in Older Adults** | | | | | | |
| Cotier et al., 2017 | Healthy controls (same sample as Shao et al., 2016). | Attention-based compassion meditation training | Length: 8 weeks (22 classes total)  Frequency: Unspecified  Duration: 1.5 hours  Supplementary: One 3-hour intensive session  Homework: Asked to practice for a minimum of 20 minutes on days without sessions. | Relaxation training | 6 min resting state  3T Philips MR scanner | Group by Time Interaction examined.  Whole brain RSFC networks constructed (1024x1024 matrix)  Global, intermediate (intra-module, inter-module), and local (i.e., nodal) level |
| Fam et al., 2020 | Mild cognitive impairment | Mindfulness awareness program (MAP) | Length: 12 weeks  Frequency: Weekly  Duration: 40 minutes  Homework: Asked to practice mindfulness at home daily. | Health education program | Duration of resting state scan not provided.  3T Siemens scanner | Group by Time interaction contrast.  Temporal efficiency analysis  Temporal RSFC networks constructed over the time-series (285 windows) using 45 ROIs |
| Li et al., 2022 | Clinical; late-life depression | MBCT+TAU | Length: 8 weeks  Frequency: Weekly  Duration: 60-90 minutes  Supplementary: Online support available.  Homework: Asked to practice for 45 minutes per day outside of sessions. | TAU | 8 minute resting state scan  3T GE EXCITE HD scanner | Group by time interaction effect.  RSFC network constructed using 116 anatomical ROIs (AAL cortical and sub-cortical atlas) |
| Sevinc et al., 2021 | Healthy controls | MBSR | Length: 8 weeks  Frequency: Weekly  Duration: 45 min practice, 15 min instruction, check in, QA).  Homework: Assigned to practice 45 minutes per day. | Cognitive fitness program | 3T Siemens Prisma | Group by time interaction effect and within group (pre vs post) contrasts examined.  Seed-based RSFC  (posteromedial cortex or pcc/rsp, hipp)  Whole brain gPPI (pre vs post) with PACC score as regressor and R hipp or L hipp as seeds. |
| Shao et al., 2016 | Healthy controls (sample the same as Cotier et al., 2017) | Attention-based compassion meditation training | Length: 8 weeks (22 classes total)  Frequency: Unspecified.  Duration: 1.5 hours  Homework: Asked to practice for a minimum of 20 minutes on days without sessions. | Relaxation training | 6 minutes resting state  3T Philips scanner | Group by time interaction examined.  Whole brain rsfc  (Seed: PCC/precuneus) |
| Wells et al., 2013 | Mild cognitive impairment | MBSR | Length: 8 weeks  Frequency: Weekly  Duration: 2-hour  Supplementary: One day mindfulness retreat  Homework: Encouraged to practice outside of class. | Within-Subject Design | Resting state scan: 6 minutes  3T Siemens | Group by time interaction effect examined.  FC between 4 ROIs:  pcc, mPFC, L hipp, R hipp |
| **Task fMRI in Younger and Middle Age Adults** | | | | | | |
| Seminowicz et al., 2020 | Clinical; patients with headache disorder or migraines | MBSR | Length: 16 weeks  Frequency: Weekly for first 8 weeks and bi-weekly for another 8 weeks  Duration: 2-hour sessions  Supplementary:  Homework: | Stress management of headaches | Multi-source interference task (MSIT) which reliably activates mid cingulate and “extrinsic mode network”  Siemens 3T Prisma Fit MRI | Primary outcome: change in activation during cognitive task in ROIs: L/R dorsal anterior insula, L/R DLPFC, anterior midcingulate cortex (selected based on activation during MSIT at baseline for group combined)  Secondary analyses: whole-brain analysis  Also conducted voxel based morphometry (VBM) of gray matter volume. |
| Turpyn et al., 2021 | Sub-clinical; stressed  highly stressed mothers of adolescents | Parenting Mindfully intervention | Length: 8 weeks  Frequency: Weekly  Duration: 2-hour sessions | Parent Education including handouts and three 30-min sessions over eight weeks. | Emotion Processing (IAPS) Task.  Siemens 3T Allegra  Resting state scan was 6-minutes. | Contrast of interest: general negative (negative > neutral image).  ROIs: amygdala, dACC, posterior insula, and anterior insula |
| Farb et al., 2010 | Non-clinical; stressed (recruited from MBSR program) | MBSR | Length: 8 weeks  Frequency: Weekly  Duration: 2.5-hour sessions  Homework: Daily homework with CD-guided and self-guided mindfulness practices. | Waitlist Control | Sadness Provocation Task.  3T General Electric Signa | Contrast: Sad film vs. Neutral film.  Whole brain followed by ROI analysis. |
| Kral et al., 2018 | Long term meditators (average of 9081 lifetime hours) and healthy controls | MBSR* | Length: 8 weeks  No other details about the intervention provided. | Active control (Health enhancement program) | Emotion Processing (IAPS) Task.  3T GE X750 | Amygdala ROIs for Negative>Neutral contrast.  Amygdala-vmPFC examined.  gPPI analysis conducted as well. |
| Desbordes et al., 2012 | Healthy controls | Mindful Attention Training | Length: 8 weeks  Frequency: Weekly  Duration: 2-hour sessions  Homework: Asked to mediate for an average of 20 minutes a day outside of class. | Cognitively-Based Compassion Training or active control | Emotion Processing (IAPS) Task.  3T Siemens Tim-Trio | ROI: L and R amygdala.  Negative, Positive and Neutral image conditions examined.  Also examined whole brain analysis. |
| Kirk et al., 2014 | Healthy controls  (2 separate samples). | Mindfulness training | Length: 8 weeks  Frequency: Weekly  Duration: 2.5-hour sessions  Supplementary: One full-day retreat.  Homework: Asked to mediate for 20 minutes on non-class days with guided audio meditations. | Progressive muscle relaxation | Secondary Reward Processing Task (Art Viewing Paradigm) and Primary Reward Processing Task (Juice delivery)  3T Siemens Trio | ROIs: Bilateral insula vmPFC and left mid/anterior insula ROIs.  gPPI using vmPFC as seed region. |
| Leung et al., 2018 | Healthy controls | Awareness-based Compassion Meditation | Length: 6 weeks  Frequency: 2 sessions per week for weeks 1-3. Home-based guided practice for weeks 4-6  Duration: Total practice time average = 543 minutes.  Supplementary: Intensive retreat for week 5 | Relaxation training | Emotion Processing (IAPS) Task  3T Philips Achieva | ROIs: L and R amygdala.  Happy, sad, and neutral conditions.  happy > neutral or sad > neutral contrast. |
| Goldin and Gross, 2010) | Clinical; social anxiety disorder | MBSR | Length: 8 weeks  Frequency: Weekly  Duration: 2.5-hour sessions  Supplementary: One half-day mediation retreat  Homework: Audio files to support home practice. | Within-Subject Design | Negative Self-Beliefs Task  3T GE Signa scanner | Initial contrasts for pre to post MM:  Distraction-focused attention versus react negative self-beliefs.  Breath-focused attention versus react negative self-beliefs.  Whole brain analysis  Then assessed BOLD signal time series in R dorsal amygdala at baseline and follow up during react negative self-belief. |
| Goldin et al., 2012 | Clinical; social anxiety disorder | MBSR | Length: 8 weeks  Frequency: Weekly  Duration: 2.5-hour sessions  Supplementary: One half-day mediation retreat  Homework: Audio files to support home practice. | Active control (aerobic exercise) | Self-Referential Encoding Task  3T GE Signa scanner | Contrasts: Negative self(versus case) and positive self(versus case)  ROIs: VMPFC, DMPFC, and PCC. Also ran whole-brain analyses.  Used Repeated-measures ANOVA with follow up t-tests.  Secondary analyses: neural temporal dynamics of 3 ROIs. |
| Williams et al., 2020 | Clinical; remission from major depressive disorder | MBCT | Length: 8 weeks  Frequency: Weekly  Duration: 2.5-hour sessions  Supplementary: One all-day practice session.  Homework: Audio files to encouraged home practice. | Within-Subjects Design | Self-Blame Task  3T Philips Achieva | Whole brain analyses.  Self-blame>other-blame, self-blame>fixation, and other-blame>fixation. |
| Braden et al., 2016 | Clinical; chronic low back pain | MBSR | Length: 4 weeks  Frequency: Weekly  Duration: 2-hour sessions  Supplementary:  Homework: Suggested assignments of 20-30 mins per day outside of class. | AC  Reading Control group  “Relaxation Techniques for Health: An Introduction” | Sadness Induction Task (Film)  3T Philips Ingenia scanner | ROIs: sgACC, Anterior Insula, dmPFC, and vlPFC.  Examined sadness condition and control condition separately. |
| Monti et al., 2012 | Clinical; breast cancer patients | Mindfulness based art therapy (MBAT) | Length: 8 weeks  Frequency: Weekly  Duration: 2.5-hour sessions  Supplementary:  Homework: | EC | Stress Task (Serial 7’s beginning at 1000)  3T Siemens Trio  8 min continuous and pseduo-continuous ASL (CASL) | 2x2 ANOVA (MBAT x EC) and (pre x post) for 5 conditions (1 of which was activation during stressor task).  Whole brain analysis |
| Allen et al., 2012 | Healthy controls | Mindfulness-style contemplative practice and “heart practice”  4 progressive modules: focused breath awareness, body scanning, compassion, and open-monitoring. | Length: 6 weeks  Frequency: Weekly  Duration: 2-hour sessions  Supplementary:  Homework: Instructed to complete 20 minutes of home practice per day | SRL | Affective Stroop Task  (number counting stroop task with IAPS images)  3T Siemens Trio | Time (pre<post) by emotion (negative>neutral) contrast.  Task(incongruent=congruent)>passive view.  Whole brain analysis |
| Huang et al., 2019 | Sub-clinical; bereaved sample | MBCT | Length: 8 weeks  Frequency: Weekly  Duration: 2.5-hour sessions  Supplementary: Extra 2-hour introduction session of “acknowledgment of grief and theory of psycho-physical reactions to loss” before standard MBCT program.  Homework: 30-40 mins of home practice per day. | Within-subjects | Stroop Task  3T Siemens PRISMA | Contrasts: pre- vs. post-MBCT for congruent and for incongruent trial.  Whole brain analysis |
| Carroll et al., 2022 | Sub-clinical; stressed teachers with work-related stress | MBSR | Length: 8 weeks  Frequency: Weekly  Duration: 2.5-hour sessions  Supplementary:  Homework: Participants were given a 100-page workbook and audio files. Encouraged to practice for 30 mins per day, 6 days per week. | HEP | Emotional Counting Stroop  3T Siemens Magnetom Trio | Task conditions (control, general negative, specific negative, general neutral, and specific neutral).  Pre vs post intervention effect examined between groups for each task condition.  Whole brain analysis |
| Hatchard et al., 2021 | Clinical; breast cancer survivors with chronic neuropathic pain | MBSR | Length: 8 weeks  Frequency: Weekly  Duration: 2.5-hour sessions  Supplementary: One full-day session.  Homework: | Waitlist Control | Emotional Stroop  3T Siemens TRIO MR scanner. | Contrasts: sensory pain – neutral; negative affect-neural;  Whole brain analysis  Follow up ROI analysis for regions showing significant main effect or interaction. |
| Tomasino et al., 2016 | Healthy controls | Mindfulness-Oriented Meditation (MOM) | Length: 8 weeks  Frequency: Weekly  Duration: 2-hour sessions  Supplementary:  Homework: Encouraged to practice daily for 30 minutes. | Within-subjects | Little Man (own-body mental transformation task) Vs. Letter Task ( non-bodily mental transformation task)  3T Philips Achieva | Little Man Task (Front versus Back) - Letter Task (Turned versus Unturned).  Whole brain analysis |
| Bachmann et al., 2018 | Clinical; ADHD combined subtype or inattentive subtype | Mindfulness awareness practice (MAP) | Length: 8 weeks  Frequency:  Duration:  Supplementary:  Homework: Daily seated meditation and exercises in everyday life. | Psychoeducation | N-Back Task  (one-back)  3T Siemens Magnetom Trio | Group by Time Interaction examined for (target-correct)-(nontarget-correct)  Whole brain analysis |

ABCMT=attention-based compassion meditation training;

AC=active control;

aDMN=anterior DMN;

ASL=

BATA=Behavioral Activation Therapy for Anhedonia;

DAN=Dorsal Attention Network;

DMN=Default Mode Network;

DMN=default mode network;

EC=education control group;

FAM=focused attention meditation;

fc=functional connectivity;

FPN=Frontoparietal Network;

GAD=Generalized Anxiety Disorder;

HEM=Health Enhancement through Mindfulness;

HER=Health Enhancement through Relaxation;

IMMTI=intensive mindfulness meditation training intervention;

MBAT=mindfulness-based art therapy;

MBAT=mindfulness-based attention training;

MBCT=Mindfulness based Cognitive Therapy;

MBHW= mindfulness-based health and wellness intervention;

MDD=Major Depressive disorder;

MdT = meditation training;

MPG= mindfulness practice group;

MT=Mindfulness Training;

PCGT=Person-Centered Group Therapy;

pdDMN=posterior dorsal DMN;

PM=Parenting Mindfully Intervention;

PMR=progressive muscle relaxation;

PTSD=post-traumatic stress disorder

pvDMN=posterior ventral DMN;

RCT=Randomized Controlled Trial;

ROI=region of interest,

rsfc=resting state functional connectivity;

RT=Relaxation Training;

RTI=relaxation training intervention;

SAL=Salience Network;

sc=structural connectivity;

sd=standard deviation;

SP=supportive therapy;

SRL=shared reading and listening;

TAU=treatment as usual, which was psychopharmacology only;

VA=Ventral Attention Network;
