# Supplementary material for: Three potential neurovascular pathways driving the benefits of mindfulness meditation for older adults
Source: Front Aging Neurosci. 2023 Jun 29;15:1207012. doi: 10.3389/fnagi.2023.1207012 (PMC10340530; doi:10.3389/fnagi.2023.1207012)
Supplement: Supplementary file 2 [file Table_2.DOCX]

Supplemental Table 2. RSFC Studies in Younger Adult Samples

| Study | Age Range | Sample | Demographics | MM Intervention | Analysis Approach | Summary and Behavioral Correlates |
| --- | --- | --- | --- | --- | --- | --- |
| Cernasov et al., 2021 | 18-50^RR^ | Clinical; anhedonia, transdiagnostic sample | **MBCT**: N=35 (24f, 11m)  Age=31.8(9.2)  Edu=not specified  Income=$59,280(51,120)  Race:  5 A  7 AA  1 NA  3 O  19 W  Ethnicity:  Hispanic or Latino 6  Not Hispanic or Latino 28  Prefer not to answer 1  **AC** (behavioral activation for anhedonia): N=38 (27f, 11m)  Age=27.9 (8.8)  Edu=not specified  Income: 85,945 (77,446)  Race:  9 A  8 AA  21 W  Ethnicity:  Hispanic or Latino 1  Not Hispanic or Latino 37 | MBCT: Up to 15 sessions.  Homework: encouraged between-session home practice | Growth curve models estimated change over time in anhedonia and in rsFC using average ROI-to-ROI connectivity within networks of interest.  Average ROI-to-ROI network connectivity using 300 ROI functional atlas.  Seed-to-voxel analyses employed for networks with significant change during ROI-to-ROI network connectivity analyses.  Edgewise ROI-to-ROI for whole-brain ROI-to-ROI analyses.  Networks Examined:  DMN (65 ROIs)  FPN (36 ROIs)  SAN (13 ROIs(  Reward network (8 ROIs) | ↓ average rsFC within the DMN and FPN over time.  Seed-to-voxel and edgewise rsFC analyses corroborated ↓within the DMN and between the DMN and FPN over time, across the sample.  Between person: higher than average FPN rsFC was related to less anhedonia across timepoints.  Within person: correlations not significant. |
| Chou et al., 2022 | - | Clinical; bipolar disorder | **MBCT**: N=22  Age=45.9(10.13)  Edu=15.5(1.58)  (neuroimaging: n=10)  **AC** (supportive psychotherapy): N=22  Age=33.4(14.45)  Edu=15.6(0.89)  (neuroimaging: n=5)  **HC**: N=22 (8f, 12m)  Age=33.32(8.93)  No race/ethnicity information | MBCT: 12 weeks (120 min weekly group sessions) | Time-series correlations between BOLD signal from DLPFC seed and every voxel in the brain.  Additionally, examined Fisher-transformed correlation between the DLPFC  and posterior cingulate cortex (PCC).  Post-hoc analyses for whole-brain also examined. | Time x Group Interaction:  MB<AC: L DLPFC-PCC  (at follow up MM but not AC showed negative correlation between DLPFC and PCC) |
| Creswell et al., 2016 | 24-54^RR^ | Subclinical; stressed job-seeking unemployed | **HEM**: N=18 (7f, 11m)  Age=37.94(10.96)  Edu:  HS or less 3  Tech 3  Some college 4  Degree 8  Race:  10 W  AA 6  A 1  1 Biracial  1 O  **HER**: N=17 (8f, 9m)  Age=41(9.55)  Edu:  HS or less 2  Tech 2  Some college 3  Degree 10  Race:  13 W  2 AA  0 A  Latino 1  1 O | 3-day intensive residential mindfulness meditation (HEM) vs. relaxation training program (HER) | Atlas Anatomical Automatic Labeling atlas (each with dilation of 1 mm): the first mask consisted of the left and right anterior cingulate and the second ROI mask consisted of the left and right middle frontal cortex.  4 ROIs:  L Middle Frontal Gyrus  R Middle Frontal Gyrus  L ACC  R ACC  DMN (PCC seed-based) to dlPFC | MM:  ↓ circulating log-transformed IL-6 at 4 months follow up (relative to baseline).  ↑ PCC-L DLPFC statistically mediated ↓ IL-6.  No change in employment between groups at follow up. |
| Dodich et al., 2019 | 19-25 | HC | **MT**: N=12 (2f,10m)  Age=21.63(2.02)  **CG**: N=30 (12f,18m)  Age=22.16(1.33)  Monolingual Italian speaking undergraduate students.  No race or ethnicity data provided. | Meditation training consisted of 4 one-hour sessions per week over 4 consecutive weeks (total of 16 hours).  No other commitments, including home practice, were requires. | Blind gICA decomposition of resting‐state fMRI data  Intrinsic brain functioning in terms of spectral power of resting‐state networks (RSNs), a measure of the coherence of intranetwork intrinsic activity (maximal for high power spectra at low frequencies). | ↑ coherence of intrinsic brain activity in bilateral DLPFC and dorsomedial PFC, dorsal ACC and bilateral fronto-insular cortex  MG: decreased power at ultra-low frequencies (<0.02Hz) and increased power in typical low-middle frequencies (<0.01Hz) |
| Fahmy et al., 2019 | 18-55^RR^ | Clinical; opiate dependence | **MM**: N=16 (1f,15m)  Age=31.8(4.2)  **TAU**: N=16 (1f,15m)  Age=30.4(5.5)  No race or ethnicity data provided. | Mindfulness-based therapy translated from English to Arabic.  Average 4 sessions a week over their admission period of one month. | Independent component analysis  DMN (aDMN and pDMN) | MBT vs TAU  anterior DMN: ↓ R IFG connectivity with MBT  Within MM-group:  ↓ R superior frontal gyrus connectivity was detected after treatment.  Within TAU group:  pDMN-  ↓ fc in R hippocampus, L precentral gyrus  aDMN:  ↑ right superior and right middle temporal gyrus  ↓ L thalamus and L pallidum |
| Farb et al., 2007 | - | Subclinical; recruited upon enrolment in the mindfulness-  based stress reduction | **MBSR**: N=20 (15f, 5m)  Age=45.55(13.38)  **WL**: N=16 (12f, 4m)  Age=42(9.24)  No race or ethnicity data provided. | MBSR: 8-week weekly MBSR course. Minimum 45 minutes practice 6 days per week. | Whole-brain time-series activation patterns were compared to activation in a volume of interest (spherical, 5 mm radius) defined in the narrative vs experiential contrast in the MT group.  Ventral and dorsal mPFC  R lateral PFC  R insula  R secondary somatosensory cortex | Controls:  R insula-vmPFC coupling  MT:  R insula and vmPFC not coupled  ↑ R insula-DLPFC |
| Gan et al., 2022 | - | HC | **MBSR**: N=16 (8f,8m)  Age=27.63 (1.25)  Edu=17.00 (0.57)  **AC** (relaxation practice): N=16 (8f,8m)  Edu=15.17 (0.67)  Age=28.06 (1.60)  Han Chinese | MBSR: 8-week program consisted of 2-hour weekly group meetings and homework. | Performed voxel-wise seed-based analyses using ROIs and ROI-to-ROI analyses.  Five spherical ROIs::  R ACC  L posterior insula  R postcentral gyrus  L medial superior frontal gyrus  L anterior insula | ↓ amplitude of low-frequency fluctuations in the R anterior cingulate gyrus, left anterior and posterior insula, and L superior medial frontal gyrus in MBSR practitioners. |
| Huang et al., 2021 | 25-66 | Subclinical; bereaved sample | N=23 (21f, 2m)  Age=48.35 (11.14)  Mandarin speakers.  No race/ethnicity data. | MBCT; 8-week program consisted of weekly 2.5-hour sessions plus recommended 45 minutes per day homework. | Used 245 ROIs to generate 11 networks:  DAN, VAN, SUB, SAL, FPN, VN, DMN, AN, CON, SMN, and MN.  4 additional ROIs for amygdala and hippocampus. | ↓ FC between DMN, FPN, SAL  ↓ internetwork connectivity within:  ↓ SAL-DMN  ↓ FPN-DMN  ↓ DMN-DMN  Behavioral rating changes in mood did not show a consistent pattern with FC:  The caudate-cingulo-opercular network (CON) change in RSFC and the sensorimotor network (SMN)-visual  network (VN) changes in RSFC show the opposite patterns with change in anxiety symptoms and emotion regulation difficulties. |
| King et al., 2016 |  | Clinical; PTSD (OEF/OIF veterans) | **MBET**: N=14  Age=32.43(7.54)  13 European Am  1 African Am  Edu:  HS 2  Some College 11  Graduate 1  **AC** (PCGT): N=9  Age=31.67 (10.14)  8 European Am  1 African Am  Edu:  HS 3  Some College 5  Graduate 1 | 16-week group therapy groups (2 hours each) for PTSD. | DMN: posterior cingulate cortex (PCC) and ventral medial prefrontal cortex (vmPFC) seeds  SAL: amygdala seeds. | MM:  ↑ DMN rsFC (PCC seed) and L DLPFC  ↑ PCC-DLPFC and ↓PTSD symptoms  Group x time interaction found MBET:  ↑ L DLPFC and dorsal ACC |
| Kral et al., 2019 | 25-65^RR^ | HC | **MBSR**: N=31 (18f, 13m)  Age=41.4(12.9)  **AC** (health education program): N=34 (22f, 10m)  Age=43.6(13.1)  **WL**: N=30 (19f, 11m)  Age=43(12)  No race/ethnicity data. | MBSR: 8 weekly group session (3 hours for first and last week, 2.5 hours for weeks 2-7) plus all day mindfulness retreat.  Homework: (45 minutes, 6 of 7 days each week) | Compute whole brain FC analysis with PCC seed and DLPFC.  Separate PCC seed-target ROIs analyses for  PCC–DLPFC.  PCC seed  PFC ROI: LPFC from prior study (Creswell et al., 2016) and anatomically defined DLPFC ROI (middle frontal gyrus). | ↑ PCC-R DLPFC for MM but not AC or WL  ↑ PCC-L DLPFC for MM but not AC  Effects did not persist at second follow up visit (5.5 months after intervention).  WM:  ↑ PCC–right DLPFC RSFC in MM was associated with increased SLF DTI from T1 to T2.  ↑ practice in MM correlated with ↑ PCC-L DLPFC  ↑ self-reported attention in MM correlated with ↑ PCC-L DLPFC |
| Mooneyham et al., 2017 |  | HC | Total: N=38 (22f, 16m)  Age=20.38 (2.28)  **MM**: n=19  **WL**: n=18  No race/ethnicity data | MBHW: 6-week program with 5.5 hours daily sessions including 60 minutes of formal meditation, 150 min of physical exercise,  30 min of structured small group discussion, and 90 min of lecture or discussion on practical strategies for cultivating mindfulness and wellness during daily life. Participants were encouraged to limit alcohol intake to no more than one drink a day, to eat a diet of primarily whole foods, and to consistently sleep at least 8 h each night. | Seed-based rsFC analysis using this insula region as the seed. | ↑ L insula-R vlPFC(R)  ↑ L insula- LMTG/STG  ↑ L insula-R vlPFC(R)  ↑ L insula- LMTG/STG  ↑ L posterior insula-R vlPFC  ↑ L posterior insula-L MTG/STG  ↑ CT in L posterior insula in MM but not WL |
| Roland et la., 2015 | 32-65 | Clinical; tinnitus | N=13 (9f, 4m)  Median age=55 years  No race/ethnicity or education data. | MBSR  Weekly 2 hours sessions for 8 weeks; asked to practice 30-45 min at home, and one 3-hr retreat | Seed-based functional connectivity analyses  FPN: L and R IFG seeds  cingulo-opercular network: ; L and R anterior operculum  DMN: PCC and ACC seeds | FPN:  ↑ L IFG-L superior temporal  ↑ L IFG-PCC  ↑ R IFG-PCC  ↑ L IFG-visual cortex  Cingulo-opercular:  ↑ L aO-L cingulate  ↑ R aO-L temporal/L parietal, R frontal (bordering ACC and middle frontal gyrus), and L superior temporal  DMN  No change in connectivity with either seed (PCC or ACC) |
| Seminowicz et al., 2020 | 18-65^RR^ | Clinical; participants with headache disorder/migraines | **Total Sample**: N=98 (89f, 9m)  Median age=36  Race:  71 W  17 AA  9 Other  Edu:  Up to some college: 20  College or more: 78  **MBSR+**: N=50  Subset had imaging (n=32-43 across 3 timepoints)  **AC** (stress management for headaches): N=48  (n=34-43 across 3 timepoints) | MBSR+: 16 weeks (weekly for first 8 weeks and bi-weekly for another 8 weeks). 2-hour sessions.  Participants were provided with audio CDs and handouts and a personal copy of Full Catastrophe Living by Jon Kabat-Zinn. | R dorsal aINS to the L DLPFC and component regions of the EMN. | No MM effect in ROIs.  Both Groups:  ↓ anterior mid cingulate volume (P=.04)  ↓ right dorsal anterior insula to cognitive task network (EMN)  Secondary Analyses: MM had  ↓ left dorsal anterior insula-PPC/precuneus  ↓ Left dorsal anterior insula-cuneus |
| Smith et al., 2021^a^ | - |  | **MBSR**: N=13f  Age=48.36 (12.82)  **WL**: N=10f  Age=56.50 (8.11)  No race/ethnicity or education data. | MBSR: 8-week program with weekly 2.5-hour sessions and one full day mindfulness retreat. | Seed to voxel with PCC seed | MM at follow up:  ↑ PCC-ACC/mPFC  ↓ PCC-bilateral precentral  ↓ PCC-right superior frontal gyrus  ↓ PCC-pons    No significant group x time interaction effect in whole brain analyses.  ↑ PCC-ACC/mPFC  And ↓ Pain  ↓ PCC-bilateral precentral  And ↓ Pain |
| Smith et al., 2021 | - | HC | **MM** (app): N=13 (10f, 3m)  Race:  W: 10  AA: 1  A: 1  O: 1  **WL:** N=9 (7f, 2m)  Race:  W: 7  A: 2  No education data. | Practice app-guided mindfulness meditation for approximately  12 min/day for 8 weeks. | Whole-brain FC analyses | Mindfulness practice time was correlated with increased connectivity between the lateral parietal cortex and the supramarginal gyrus.  ↑ R lateral parietal hub to vertices within hub network (L/R supramarginal gyri, L/R lateral visual cortices, L IFG, & posterior left superior temporal  Gyrus)  ↑ L posterior parietal hub to vertices in network (R/L nucleus accumbens)  MM at 8 weeks:  Changes in spectral power at several frequencies (<6mHz) in right LP and SMG |
| Su et al., 2016 | - | Pain afflicted group and HC | Total: N=34 (25f, 10m)  Age=38.59  **Pain** afflicted group: N=18  **HC**: N=16  Native speakers of Mandarin Chinese.  No race/ethnicity or education data. | MBSR: Six 2.5-h weekly plus one 8-h retreat session. | FC between Dorsal ACC and anterior insula seeds | ↑ AIC-daMCC was observed in the post-training pain afflicted group.  ↑AIC-daMCC correlated with ↓ self reported pain in pain group. |
| Tang et al., 2013 | - | Subclinical; college students interested in stress reduction (cigarette smokers and nonsmokers) | Total: N=50  Age=21.46(3.08)  Cigarette Smokers: N=27  Nonsmokers: N=33  **IBMT**: N=33 (15 smokers; 4f, 11m)  **AC** (relaxation training): N=27 (12 smokers; 4f, 8m)  No race/ethnicity or education data. | IBMT: 30 min sessions for 2 weeks (5 hours training in total). | Whole-brain fractional ALFF | MM>RT in smokers at follow-up:  ↑ activity at ACC/mPFC  ↑ activity at IFG/vlPFC  ↓ PCC/precuneus, cerebellum  At baseline:  Smokers<HC  ↓ activity in ACC, left lateral PFC  ↓ craving in MM but not RT group among smokers |
| Tang et al., 2017 | - | HC | N=25 (13m, 12 f)  Age=21(1.6)  No race/ethnicity or education data. | IBMT: 30 min sessions for 2 weeks (5 hours training in total). | Whole brain FC based on 116 labeled ROIs from automated anatomical labeling (AAL) atlas.  Classifiers trained on measures of functional connectivity to reliably differentiate (with 72% accuracy) patterns of connectivity from before vs. after the IBMT training. | ↑ fc (60 connections), primarily involving bilateral superior/middle occipital gyrus, bilateral frontal operculum, bilateral superior temporal gyrus, right superior temporal pole, bilateral insula, caudate and cerebellum. |
| Taren et al., 2015 | - | Subclinical; stressed unemployed community adults | Total: N= 35  **MM**: N=18 (7f, 11m)  Age= 37.94 (10.96)  Race:  W: 10  AA: 6  A: 1  Edu:  HS or Less: 3  Tech: 3  Some college: 4  Degree: 8  **RT**: N=17 (8f, 9m)  Age=41.00 (9.55)  Race:  W: 13  AA: 2  A: 0  O: 1  Edu:  HS or Less: 2  Tech: 2  Some college: 3  Degree: 10 | 3-day intensive residential mindfulness meditation (HEM) vs. relaxation training program (HER) | Whole brain rsFC with L and R amygdala seeds for rsFC | MM but not RT:  ↓ right amygdala-sgACC rsFC  Whole brain analysis at baseline between stress and rsfc:  ↑ amygdala-sgACC with ↑ stress  At follow up:  ↓ right amygdala-sgACC fc  ↓ hair cortisone with trend at 4-month follow-up |
| Taren et al., 2017 | - | Subclinical; stressed unemployed community adults | Total: N= 35  **MM**: N=18 (7f, 11m)  Age= 37.94 (10.96)  Race:  W: 10  AA: 6  A: 1  Edu:  HS or Less: 3  Tech: 3  Some college: 4  Degree: 8  **RT**: N=17 (8f, 9m)  Age=41.00 (9.55)  Race:  W: 13  AA: 2  A: 0  O: 1  Edu:  HS or Less: 2  Tech: 2  Some college: 3  Degree: 10 | 3-day intensive residential mindfulness meditation (HEM) vs. relaxation training program (HER) | Seeded RS BOLD fMRI images were applied in a group-level flexible factorial analysis with two factors specified, time (pre- and post-intervention) and group (HEM vs HER groups).  RS networks involved in attention/executive functioning (middle frontal cortex, inferior frontal cortex, superior and posterior parietal lobule, middle temporal cortex) | ↑ Left dlPFC FC to the right inferior frontal gyrus, R middle frontal gyrus, R supplementary eye field, R superior/posterior parietal cortex and L middle temporal gyrus  ↑ Right dlPFC showed increased connectivity to right middle frontal. |
| van der Gucht et al., 2020 | 36-55 | Clinical; breast cancer survivors who reported cognitive impairment | 45% finished secondary school,  45% held a higher education degree  10% never finished secondary school  **MM**: N=18  Age: 43.89 (6.03)  **WL**: N=15  Age=47.4 (5.45)  No race/ethnicity data. | MBSR and MBCT for patients with cancer combined. Delivered four 3-hour, in-person group sessions spread over 8 weeks and in between online support. | ROI-to-ROI FC maps  DMN: medial PFC, R/L lateral parietal cortex, PCC  SAL: ACC, R/L anterior insula, R/L rostral PFC, R/L supramarginal gyrus  DAN: R/L FEF, R/L intraparietal sulcus,  FPN: L/R lateral PFC, R/L posterior parietal cortex | ↑ ACC-L IPS  ↑ ACC-R IPS  Increased FC between DAN and SAL.  Significant improvements emotional distress and fatigue.  Improved distress related to FC change between ACC and left IPS. |
| Xiao et al., 2019 | - | HC | **MBSR**: N=16 (8f,8m)  Age=27.63 (1.25)  Edu: 17.00 (0.57)  **AC**: N=16 (8f,8m)  Age=28.06 (1.60)  Edu=15.17 (0.67)  Han Chinese | MBSR: 8-week program with 2-hour weekly session.  Homework: 30 minutes of meditation practice. | ReHo analysis  DMN  aDMN (L/R ACC, L superior frontal cortex/medial orbital, L PCC)  pvDMN (R/L precuneus; L PCC, R median cingulate cortex)  pdDMN (L/R precuneus, L cuneus) | MBSR (post)  ↑ regional homogeneity in the right superior parietal lobule (extending into right supplementary motor area and mid-cingulate)  ↑ regional homogeneity in left postcentral gyrus (extending into precentral gyrus)  ↓ ReHo at FU for L PCC & R/L precuneus (i.e., pdDMN)  ↓ ReHo at FU for left cuneus  ↓ left postcentral gyrus-R precuneus  ↓ left postcentral gyrus-R superior frontal medial gyrus  ↑ left postcentral gyrus-midcingulate  ↑ left postcentral gyrus-precentral  ↑ left postcentral gyrus-insula |
| Xue et al., 2011 | - | HC | N=32 (17f, 15m)  Age=21.44 (1.59)  **IBMT**: N=15 (8f, 7m)  **AC** (relaxation training): N=17 (9f, 8m)  Native speakers of Chinese  No race/ethnicity or education data. | IBMT: 4-weeks of Monday-Friday 30 minutes daily practice sessions (11 hours of training total). | Whole brain FC analysis with anatomical automatic labeling with 90 nodes.  Use graph metrics: regional topology of nodal efficiency and degree | ↑ in the network efficiency and connectivity of the ACC |
| Yang et al., 2016 | - | HC | N=13 (10f, 3m)  Age= 24.53(5.89)  Native Spanish speakers  No race/ethnicity or education data. | Mindfulness Training:8-week program with weekly 1.5-hour sessions.  Homework: 45 minutes of daily practice. | Seed-based FC, Group ICA, and ReHo analyses.  Pregenual ACC  dACC  DMN | ↓ depression/anxiety scores  ↓ pregenual anterior cingulate-L PCC/precuneus, L dmPFC, R superior temporal gyrus, L middle occipital gyrus, L inferior temporal gyrus  ↑pregenual ACC- R inferior temporal gyrus, R IFG, and R TPJ/IPL  ↓dACC-calcarine sulcus and cuneus  ↑dACC-cerebellum, R inferior parietal lobule, PCC  No changes to DMN ICA.  No ReHo changes. |
| Yang et al., 2019 | - | HC | N=14 (10f, 4m)  Age=24.53 (5.90)  Native Spanish speakers  No race/ethnicity or education data. | Mindfulness Training:8-week program with weekly 1.5-hour sessions.  Homework: 45 minutes of daily practice. | Whole Brain ALFF | ↑ CT of L Precuneus and L superior parietal lobule.  ↓ ALFF in left PCC/precuneus correlates with the reduction of depression scores. |
| Zamoscik et al., 2020 | - | Clinical; emotionally challenged remitted depressed patients | **MBAT**: N=24 (16f, 8m)  Age=37.75 (9.66)  Edu:  8 CSE or high school diploma  16 A levels  **AC** (progressive muscle relaxation): N=25 (16f, 9m)  Age=39.24 (11.70)  Edu:  7 CSE or high school diploma  18 A levels  No race/ethnicity data | MBAT: 4-week program held once per week (50min, 5 individual sessions) plus audio-guided daily homework (20min/day). | Whole brain rsFC  DMN (seeds in PCC and parahippocampus) | ↓ respiration pattern variability was related to ↓ anxiety.  No changes in DMN connectivity after training. |
| Zhang et al., 2021 | 19-20 | HC | N=11 (5 f, 6m)  Age: 19.09 (0.54)  No race/ethnicity or education data | Focused attention meditation (FAM): 2 months with 15 min meditation once or twice per week during class. Required to practice FAM for at least 10 min per session and no less than 5 times per week. | Seeds from DMN (PCC and vmPFC) and DAN (L/R middle  temporal area, L/R frontal eye field, L/R superior parietal lobule)  Dynamic arterial spin labeling | FAM practice time correlated with  ↑ rsFC between L superior parietal lobules and mPFC.  ↑ rsFC between DMN and DAN, DAMN and insula, and DAN and FPN  ↓ rsFC between DMN and FPN and DAN and visual regions. |
| Zhang et al., 2021(a) | 19-20 | HC | N=10 (4f, 6m)  No race/ethnicity or education data. | Focused attention meditation (FAM): 2 months with 15 min meditation once or twice per week during class. Required to practice FAM for at least 10 min per session and no less than 5 times per week. | BOLD signal time series FC maps and voxel-based morphometry analysis.  Five ROIs from the DMN (PCC), DAN (LSPL and RSPL), and VC (LMT and RMT). | Practice time correlated with changes of rsFC between the DMN and DAN, between DMN and FPN but negatively associated with changes of rsFC insula, and between DAN and the between DMN and FPN, and between DAN and visual regions.  No gray matter changes. |
| Zhao et al., 2019 | - | Clinical; generalized anxiety disorder | N=32 (24f, 6m)  Age=33.62 years (7.71)  Edu- 16 years (2.97).  Ethnicity: Han Chinese. | MBCT: 8 weeks  Home practice: at least 30 min | ReHo analysis  FC of DMN: voxel wise using PCC seed and region of interest-wise: overlay between DMN regions and ReHo results | T2 to T3  ↓ReHo in broad regions of the limbic system (R middle temporal gyrus, bilateral insula, bilateral superior temporal gyrus, R median cingulate gyrus, and L hippocampus)  ↑ ReHo in L temporal pole  ↑DMN functional connectivity in the anterior cingulate cortex (ACC) and bilateral insula.  Overlapping regions of reduced ReHo and increased DMN functional connectivity were observed in the mid-cingulate cortex (MCC) and bilateral insula.  The increased PCC-ACC and PCC-insula functional connectivity following MBCT were related to anxiety improvements. |
| Zimmerman et al., 2019 | - | Clinical; tinnitus | N=12 (7f, 5m)  Mean age=51.42 (10.63)  No race/ethnicity or education data. | MBCT: 8-week program consisting of weekly 2 hours sessions.  Homework: asked to practice 40-60 min daily | Seed-to-voxel whole brain connectivity analyses.  DMN: mPFC and PCC seeds  DAN  AMG: bilateral amygdala seed  FPN  CON  Used Graph Metrics | ↓ FC among DMN, cingulo-opercular network, and amygdala across intervention.  No differences in FC with seeds in DAN or FPN and rest of the brain.  ↓ mPFC/PCC-R thalamus and L calcarine sulcus  Further, only rsFC between the brain and the amygdala, DAN, and FPN significantly predicted tinnitus severity symptoms. |

A=Asian; AA=African American; AC=active control; ADHD=attention-deficit/hyperactivity disorder; aDMN=anterior DMN; AMG=amygdala; CON= control network; DAN=Dorsal Attention Network; DMN=Default Mode Network; Edu=education; f=female; FAM=focused attention meditation; fc=functional connectivity; FPN=Frontoparietal Network; GAD=Generalized Anxiety Disorder, HC=Healthy Controls; HEM=Health enhancement through Mindfulness; HER=Health enhancement through Relaxation; HS=High school; ICA=independent component analyses; IMMTI=intensive mindfulness meditation training intervention; l=left; m=male; MBAT=mindfulness-based attention training; MBCT=Mindfulness based Cognitive Therapy; MBHW= mindfulness-based health and wellness intervention; MDD=Major Depressive disorder; MdT=meditation training; MM=mindfulness meditation; MPG=mindfulness practice group; MT=Mindfulness Training; N=sample size; NS=not significant; O=Other; OEF/OIF=Operation Enduring Freedom/Operation Iraqi Freedom; PTSD=post-traumatic stress disorder; PCGT=Person-Centered Group Therapy; pdDMN=posterior dorsal DMN; PMR=progressive muscle relaxation; pvDMN=posterior ventral DMN; R=right; RCT=Randomized Controlled Trial; ReHo=Regional Homogeneity Analysis ROI=region of interest; RR=recruitment range; rsfc=resting state functional connectivity; RT=Relaxation Training; SAL=Salience Network; sc=structural connectivity; SES=socioeconomic status; SP = supportive therapy; VA=Ventral Attention Network; W=White; WC=Waitlist control.
